# A validation and extended description of the Lund taxonomy for urothelial carcinoma using the TCGA cohort

**DOI:** 10.1038/s41598-018-22126-x

**Published:** 2018-02-27

**Authors:** Nour-al-dain Marzouka, Pontus Eriksson, Carlos Rovira, Fredrik Liedberg, Gottfrid Sjödahl, Mattias Höglund

**Affiliations:** 1 Division of Oncology and Pathology, Department of Clinical Sciences, Lund University, Lund, Sweden; 2 Division of Urological Research, Department of Translational Medicine, Lund University, Skåne University Hospital, Malmö, Sweden

## Abstract

Global gene expression analysis has been a major tool for urothelial carcinoma subtype discovery. This approach has revealed extensive complexity both in intrinsic features of the tumor cells and in the microenvironment. However, global gene expression cannot distinguish between gene expression signals originating from the tumor cells proper and from normal cells in the biopsy. Here, we use a large cohort of advanced urothelial carcinomas for which both gene expression data and extensive immunohistochemistry are available to create a supervised mRNA expression centroid classifier. This classifier identifies the major Lund taxonomy tumor cell phenotypes as defined by IHC. We apply this classifier to the independent TCGA dataset and show excellent associations between identified subtypes and genomic features. We validate a progressed version of Urothelial-like A (UroA-Prog) that shows *FGFR3* mutations and *CDKN2A* deletions, and we show that the variant Urothelial-like C is almost devoid of *FGFR3* mutations. We show that Genomically Unstable tumors are very distinct from Urothelial-like tumors at the genomic level, and that tumors classified as Basal/SCC-like all complied with the established definition for Basal/SCC-like tumors. We identify the Mesenchymal-like and Small-cell/Neuroendocrine-like subtypes, and demonstrate that patients with UroB and Sc/NE-like tumors show the worst overall survival.

## Introduction

Molecular classification of bladder cancer has been a central issue during recent years, and it has become increasingly clear that this tumor type is a far more complex disease than previously recognized^1,2^. So far, four classification systems have been described. Damrauer *et al*.^3^ presented a two-tiered system, later extended to include three classes, the Luminal, Basal, and Claudin-low classes^4^. MDA presented a three-tiered system, later extended to include five classes, the Luminal, Luminal-p53, Basal, Basal-p53 classes, and the “double negative” class of tumors^5,6^. TCGA produced a four-class system with groups referred to as I-IV^7^ while the Lund group presented a six-class system based on global mRNA expression, Urothelial-like, Genomically Unstable, Epithelial Infiltrated, SCC-like/Mes-like, SCC-like/UroB, and Sc/NE-like^8^. Most published investigations are based on tumor biopsies from muscle-invasive tumors. However, Hedegaard *et al*.^9^ defined three major molecular subtypes of non-muscle-invasive tumors, and the original Lund classification cohort^10^ was composed of two thirds non-muscle-invasive tumors. Classification of muscle-invasive tumors poses a special problem because they are to large extent communities of tumor, immunological and stromal cell types. Consequently, it is difficult to make definite statements on the existence of particular *tumor cell phenotypes* based on global gene expression analyses only. To resolve this discrepancy, Sjödahl *et al*.^11^ assembled a series of >300 advanced tumors from which both global gene expression analyses (mRNA) and tissue microarrays (TMA) were generated. By the use of extensive immunohistochemical (IHC) analyses and a large panel of protein markers Sjödahl *et al*.^11^ showed that tumors with specific *tumor cell phenotypes* may diverge or converge with respect to gene expression clusters. In most cases the divergence/convergence could be attributed to infiltrating non-tumors cells. This challenges the objectives for classification and question what a given tumor subtype name signify; a tumor cell phenotype or a community of cells. In the present investigation, we start by constructing an mRNA classifier that captures the full complexity of the Lund Taxonomy IHC defined tumor cell phenotypes, including their respective immune and stromal infiltrated counterparts. The classes include Urothelial-like (UroA-Prog, UroB, UroC), Genomically Unstable (GU), Basal/SCC-like, Mesenchymal-like (Mes-like), and the Small-cell/Neuroendocrine-like (Sc/NE-like) subtypes^11^. We apply this classification scheme to the independent TCGA cohort of mainly muscle-invasive tumors, and find an excellent association with previously described class defining gene signatures, gene mutations, genomic alterations, and expression of transcription factors. This validates and demonstrates the biological relevance of the Lund Taxonomy.

## Results

### Deriving a tumor cell phenotype oriented mRNA based classifier for bladder cancer

**Figure 1:**
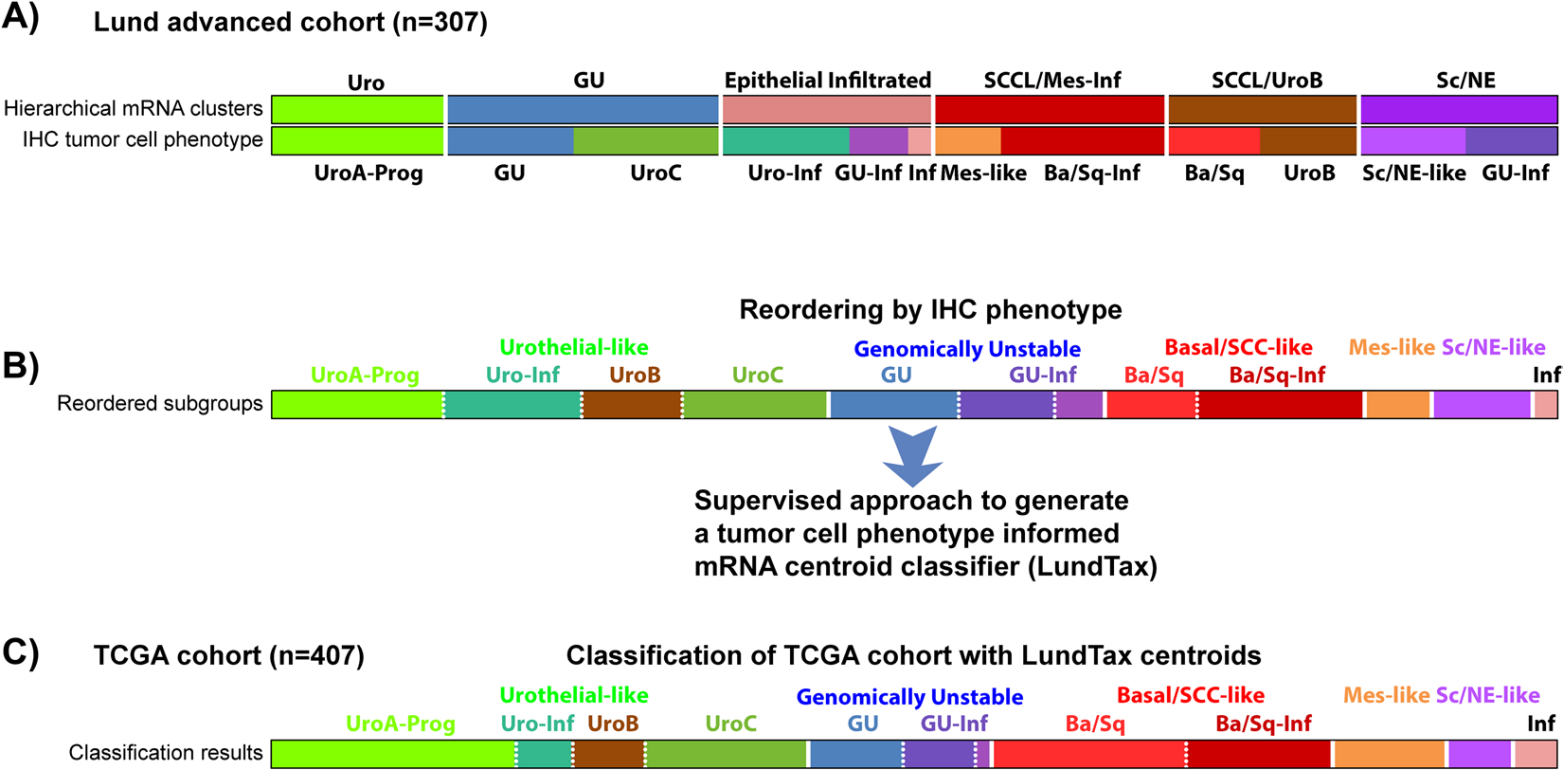
Classification of the TCGA cohort into LundTax tumor cell phenotypes. (**A**) The Lund advanced bladder cancer cohort (n = 307) was first grouped by global gene expression profiling. The samples were then stratified further into specific tumor cell phenotypes using IHC with antibodies for 25 proteins^11^. Top panel: Hierarchical clustering on global mRNA data. Bottom panel: Schematic view of how tumor cell phenotypes relate to the respective mRNA cluster. (**B**) The same data as in panel A but reordered based on tumor cell phenotype. Using this grouping and mRNA gene expression data, a 12-group tumor cell phenotype centroid classifier (LundTax) was produced. (**C**) The TCGA bladder cancer dataset (n = 407) arranged according to the LundTax centroid classifier.

To arrive at a *tumor cell phenotype* directed mRNA based classifier, we made use of previously published data^11^ for which both mRNA profiling and IHC analyses were available. In this previous study, we first grouped tumor cases according to global gene expression profiling, and then applied extensive IHC using antibodies for twenty-five proteins to refine the classification. This revealed several discrepancies between the actual *tumor cell phenotypes*, as determined by IHC, and their grouping by global gene expression profiling. Thus, tumors of identical tumor cell phenotypes could end up in different gene expression clusters, whereas in other cases, tumors of different tumor cell phenotypes would form a single gene expression cluster. We refer to this as divergence and convergence at the global gene expression level. Here we use a supervised classification approach on the Sjödahl *et al*. data to construct an mRNA-based classifier to identify IHC determined *tumor cell phenotypes* of urothelial carcinoma (Fig. 1, Supplementary Fig. 1). This approach differs from standard procedures where unsupervised clustering methods are applied to global gene expression to identify groups of tumors. This new mRNA-based classifier, the LundTax classifier, accomplishes the same as extensive IHC analyses and is hence *tumor cell phenotype* directed. The classifier was trained to identify the Urothelial-like (Uro), including the related UroA-Prog, UroB, and UroC, the Genomically Unstable (GU) and Basal/SCC-like (Ba/Sq), the less frequent Mesenchymal-like (Mes-like), and Small-cell/Neuroendocrine-like (Sc/NE-like) subtypes, as well as their infiltrated counterparts. To validate that this classifier *de facto* identifies biologically relevant and coherent tumor cell phenotypes, we applied it to the independent 407 TCGA samples of advanced urothelial carcinomas. The TCGA cohort was, accordingly, classified into 42% Uro, with the subgroups UroA-Prog, UroB, UroC and Uro-Inf, into 14% GU with the subgroups GU and GU-Inf, into 27% Basal/SCC-like with the subgroups Ba/Sq and Ba/Sq-Inf, into 9% Mes-like, and to 5% Sc/NE-like. In addition, a small group, 3%, too infiltrated to be classified was identified. This LundTax classification of the TCGA data is the basis for the subsequent analyses.

### Validation of the LundTax classification in the TCGA data using gene expression data

**Figure 2:**
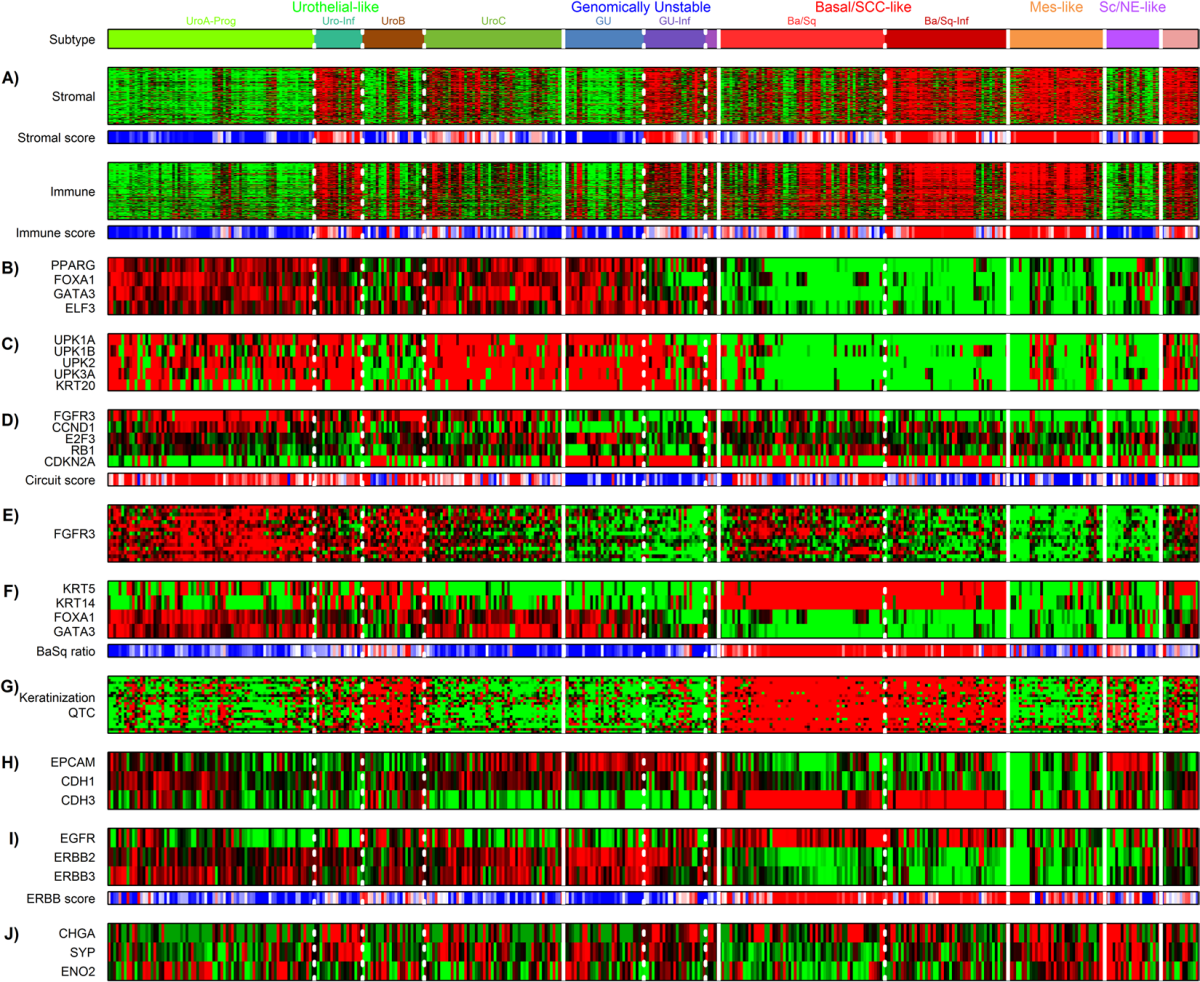
Validation of the LundTax classifier in the TCGA cohort. Cases in the TCGA cohort were grouped according to the LundTax classifier. Eleven gene signatures were then used to identify different tumor related features. (**A**) Stromal and immune cells signatures. Stromal and immune score bars show the estimated scores for tumor purity based on the ESTIMATE tool^12^. High score (red) indicate high-level infiltration; low score (blue) indicate low-level infiltration. (**B)** Transcription factors involved in urothelial cell differentiation^13^. (**C)** Cytokeratin and uroplakin genes^10^. (**D)** Genomic circuit genes^14^ that distinguish the GU subtype from the Uro subtype. Circuit ratios were calculated for each sample as *RB1* + *FGFR3* + *CCND1* − *E2F3* − *CDKN2A* using log2 mRNA expression values. High ratio (red) indicate Uro, low ratio (blue) indicate GU. (**E)** The *FGFR3* gene expression signature^10^. (**F)** Expression of the *KRT5*, *KRT14, FOXA1*, and *GATA3* genes, defining the Basal/Squamous-like (Ba/Sq) molecular subtype^16^. Ba/Sq ratios were calculated for each sample as *KRT5* + *KRT14* − *FOXA1* − *GATA3* using log2 mRNA expression values. High ratio (red) indicate a Ba/Sq subtype, low ratio (blue) indicate non-Ba/Sq subtype. **(G)** Keratinization gene signature^13^. (**H)** The cell adhesion genes *EPCAM*, *CDH1*, and *CDH3*. (**I)** The expression of the receptor tyrosine kinases *EGFR*, *ERBB2* and *ERBB3*. *ERBB* ratios were calculated for each sample as *EGFR-ERBB2***-***ERBB3* using log2 mRNA expression values high ratio (red) indicate EGFR expression >ERBB2 and ERBB3 expression, low ratio (blue) indicate the opposite. Gene expression color codes; red, high expression; green, low expression. Vertical lines separate major molecular subtypes; dotted lines separate subgroups of subtypes. Subtype abbreviations as in Fig. 1.

We used previously established subtype specific gene signatures to validate the centroid classification of the TCGA cohort. As infiltrating non-tumorous cells affect the overall gene expression profiles, we first examined established stromal and immune signatures^12^. As expected, subgroups classified as infiltrated, indicated with the suffix “Inf” in Fig. 2, show the strongest infiltration by stromal and immune cells as determined by the strength of the respective gene signatures. We next used two established gene signatures for urothelial-cell differentiation specifically expressed by Uro and GU tumors^11,13^. The first signature includes the regulatory factors *PPARG*, *FOXA1*, *GATA3*, and *ELF3* (Fig. 2B), the second includes the differentiation genes *UPK1A*, *UPK1B*, *UPK2*, *UPK3A*, and *KRT20* (Fig. 2C). These signatures indicate a correct classification of Uro and GU cases by the classifier. UroB shows lower levels of the differentiation genes, consistent with biological progression towards a Basal/SCC-like tumor cell phenotype^10^. We applied a previously published scoring system based on the expression of *FGFR3*, *CCND1*, *E2F3*, *RB1*, and *CDKN2A* to distinguish Uro from GU cases (Fig. 2D)^11,14,15^. The Uro and GU subtypes, as identified with the classifier, showed the expected subtype specific scores, high in Uro and low in GU tumors. Supporting this conclusion is the expression of the *FGFR3* gene signature composed of genes associated with *FGFR3* gene mutations, known to show high expression in Uro and low expression in GU cases^10^ (Fig. 2E).

We then used the consensus definition for the Basal/SCC-like subtype, *KRT5* and *KRT14* high, and *FOXA1* and *GATA3* low expression, to identify the Basal/SCC-like cases^16^. The *KRT5* and *KRT14*/*FOXA1* and *GATA3* expression ratio clearly identified the category of tumors classified as Ba/Sq and Ba/Sq-Inf by the LundTax centroid classifier as being Basal/SCC-like (Fig. 2F). The same tumors also express specific genes involved in keratinization, further validating the results of the classifier^13^ (Fig. 2G). In addition, the Ba/Sq and Ba/Sq-Inf tumors express *CDH3* (P-Cadherin)^11^ and *EGFR*, but not *ERBB2* or *ERBB3*^17^ further supporting this conclusion (Fig. 2H and I). The most characteristic feature of the Mes-like group of tumors is tumor cell expression of *VIM* and *ZEB2*, markers for a mesenchymal phenotype. However, as stromal cells also express high levels of *VIM* and *ZEB2*, mRNA analysis of whole biopsies are uninformative in this case. Still, the Mes-like group differs from both Uro and GU tumors by showing low expression of *FOXA1* and *GATA3*, and from the Basal/SCC-like group by showing low *KRT5* and *KRT14* expression (Fig. 2F). Similarly, Sc/NE-like cases show low expression of *KRT5*, *KRT14*, *FOXA1*, and *GATA3*, but differ from Mes-like subtype by rarely being infiltrated by immune or stromal cells and by expressing *EPCAM* (Fig. 2H). The classified Sc/NE tumors also showed high expression of *CHGA*, *SYP*, and *ENO2* (Fig. 2J). Taken together, by applying previously described subtype specific gene signatures, we show that the tumor cell phenotype trained LundTax classifier identifies established molecular subtypes of urothelial carcinoma at high precision.

### LundTax classification of the TCGA cohort in relation to exome mutation data

**Figure 3:**
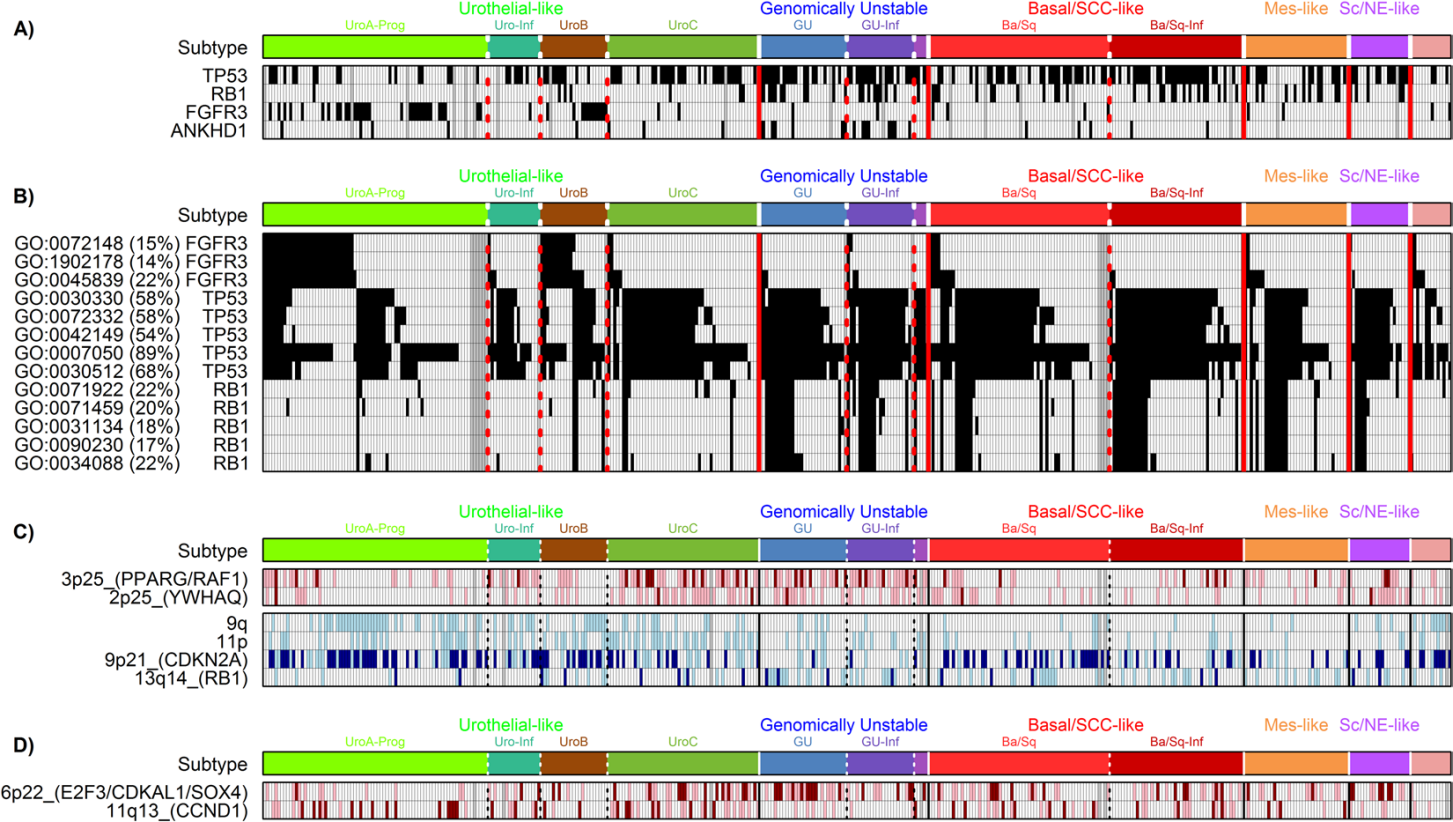
Genomic profiles of LundTax molecular subtypes in the TCGA cohort. (**A)** Differentially mutated genes. Black, mutation; white, wild type; gray, no data. (**B)** Selected differentially mutated biological processes among molecular subtypes. Three major biological processes were identified, FGFR3 signaling, apoptotic signaling pathway by TP53, and processes involved in genomic instability including RB1. Percentages in parentheses indicate the fraction of the mutated samples in the whole cohort for the given GO term. GO terms; GO:0072148, epithelial cell fate commitment; GO:1902178, fibroblast growth factor receptor apoptotic signaling pathway; GO0045839, negative regulation of mitosis; GO:0030330, DNA damage response signal transduction by p53 mediator; GO:0072332, intrinsic apoptotic signaling pathway by p53 class mediator; GO:0042149, cellular response to glucose starvation; GO:0007050, cell cycle arrest; GO:0030512, negative regulation of transforming growth factor beta receptor pathway; GO:0071922, regulation of cohesion localization to chromatin; GO:0071459, protein localization to chromosomes, centromeric region; GO:0031134, sister chromatid bi-orientation; GO0090230, regulation of centromere complex assembly; GO:0034088, maintenance of mitotic sister chromatid cohesion. (**C)** Selected genomic imbalances significantly (p < 0.05, Bonferroni corrected) associated with molecular subtypes. Light brown, gain; dark brown, amplification; light blue, loss; dark blue, homozygous loss. (**D**) Distribution of 6p22 (*E2F3, CDKAL1, SOX4*) and 11q13 (*CCND1*) amplifications across the molecular subtypes. Color codes as in panel C. Subtype abbreviations as in Fig. 1. Vertical lines, separate major molecular subtypes; dotted lines, separate subgroups of subtypes.

**Figure 4:**
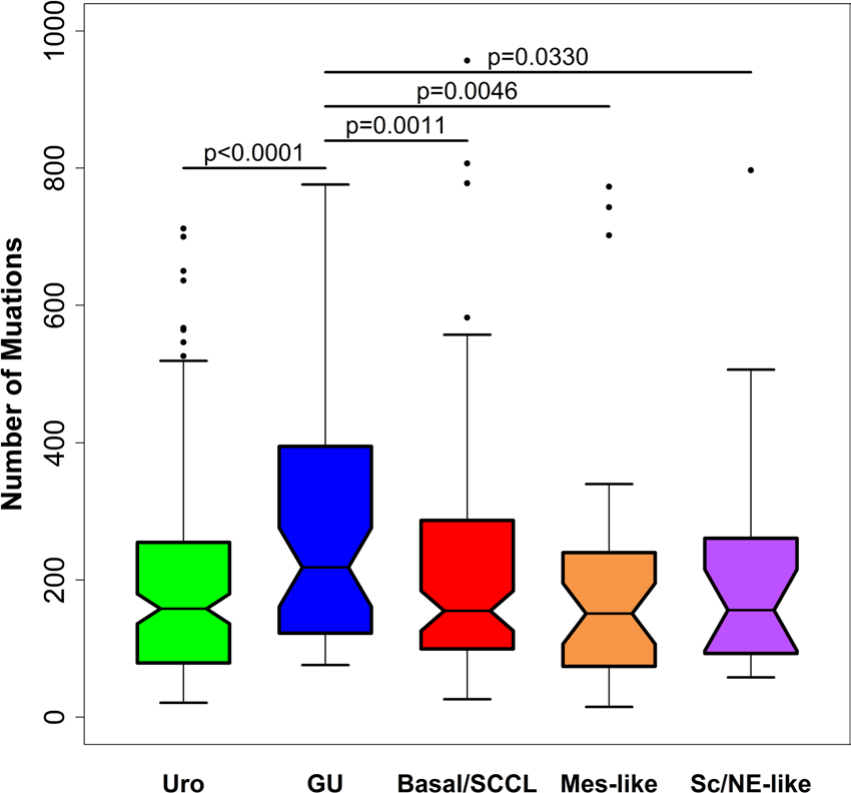
Mutational burden in LundTax bladder cancer subtypes. Mutational burden based on non-silent mutations in the major LundTax subtypes in the TCGA bladder cancer cohort (n = 389).

We identified mutations in 351 genes present in more than 5% of the 389 TCGA cases. Fisher’s exact test for association with the major classes of tumors, Urothelial-like, Genomically Unstable, Basal/SCC-like, Mes-like, and the Sc/NE-like identified four significant genes *TP53*, *RB1*, *FGFR3* and *ANKHD1* (Fig. 3A). *TP53* mutations were seen in all subtypes but were particularly enriched in the GU subtype, present in 71% of the cases. *RB1* mutations were also enriched in the GU subtype, seen in 37% of the cases, but also frequent in Basal/SCC-like tumors. *FGFR3* mutations were predominately seen in UroA-Prog (44%) and UroB (50%) cases, but were almost depleted in the UroC subtype (4%). The MutSigCV algorithm identified 30 significantly mutated genes (Supplementary Fig. S2). Fishers exact test of the MutSigCV significant genes did not yield any additional subtype specific associations. Mutations in the *ANKHD1* gene did not pass the MutSigCV test. We then formed groups of genes based on GO-terms for “biological processes”, identified tumors with mutations in any or none of the GO-term grouped genes, and then performed Fisher’s exact test for subtype associations. Mutations in a total of 50 GO-term defined processes were found to be associated with subtype (p < 0.05, Bonferroni corrected) (Supplementary Table S2). Biological processes related to fibroblast growth factor receptor (FGFR) signaling were enriched in the Urothelial-like subtypes, whereas processes related to apoptotic signaling were depleted (Fig. 3B). Processes related to chromosomal instability were enriched in GU, Basal/SCC-like, Mes-like and Sc/NE-like cases, and almost absent in the Urothelial-like subtypes. We then compared the mutational burden between the major subtypes using the complete non-silent mutation data (Fig. 4), and found GU to have the highest mutational burden. Taken together, gene mutations and GO-term associated patterns of gene mutations are distinctly associated with different molecular subtypes. Furthermore, the gene mutation data clearly delineate the Urothelial-like and the Genomically Unstable subtypes as two separate entities. The UroC differed from other Urothelial-like subtypes in one important respect, the absence of *FGFR3* mutations.

### LundTax classification of the TCGA cohort in relation to genomic data

We produced genomic imbalance maps based on TCGA copy number data for 405 cases and performed Fisher’s exact test to identify genomic imbalances associated with the major subtypes. Seven gained/amplified regions 1q21, 1q23, 2p25, 3p25, 5p, 17q11, and 18p11, and five losses/homozygous losses, 9q, 9p21, 11p, 13q14 and 22q13 showed significant association with the LundTax subtypes (Supplementary Fig. S3). The 2p25 and 3p25 segments were frequently amplified in UroC and GU cases (Fig. 3C). The 2p25 amplicon includes the *YWHAQ* gene that shows strong correlated expression with amplification (Supplementary Fig. S4). The 3p25 amplicon contains the genes *PPARG* and *RAF1*. Both *PPARG* and *RAF1* showed increased expression when amplified, however, whereas *RAF1* expression was strictly associated with gene copy changes, *PPARG* expression was not. Notably, several of the cases with amplified *PPARG* showed downregulation of the gene (Supplementary Fig. S4). Amplification of the 6p22 region showed a lower level of significance, p < 0.005 not significant after Bonferroni correction, but was particularly frequent in the UroC (24%), GU (26%), and the Sc/NE-like (29%) subtypes (Fig. 3D). The 6p22 amplification has been extensively described and includes almost invariably the *E2F3, CDKAL1, SOX4* genes^18^. Genomic amplifications of 11q13 (*CCND1*) showed borderline significance, were associated with the Urothelial-like subtypes (seen in 16%), were almost absent in GU (<2%) (Fig. 3D), and showed a strong association with *CCND1* expression (Supplementary Fig. S4). Loss of the long arm of chromosome 9 (9q) and the short arm of chromosome 11 (11p) was significantly associated with the Uro subtypes (Fig. 3C). Homozygous losses of *CDKN2A* (9p21) were particularly frequent in the Uro subtypes, seen in 39% of the cases, but also seen in Basal/SCC-like and Mes-like and Sc/NE-like tumors. However, GU tumors were almost devoid of homozygous *CDKN2A* losses, only seen in 5% of the cases (Fig. 3C). *RB1* (13q14) losses occurred predominantly in GU (44%) and Basal/SCC-like cases (28%) (Fig. 3C). Taken together, well-known and frequently occurring genomic alteration in Urothelial carcinoma show a very strong association with the different molecular subtypes. In addition, both Urothelial-like subtypes and Genomically Unstable cases show distinct genomic profiles; Uro-cases show frequent 9q, 9p21, and 11p losses and 11q13 amplifications whereas GU show frequent *RB1* losses but almost complete absence of *CDKN2A* homozygous losses.

### LundTax classification of the TCGA cohort in relation to DNA-binding transcription factors

**Figure 5:**
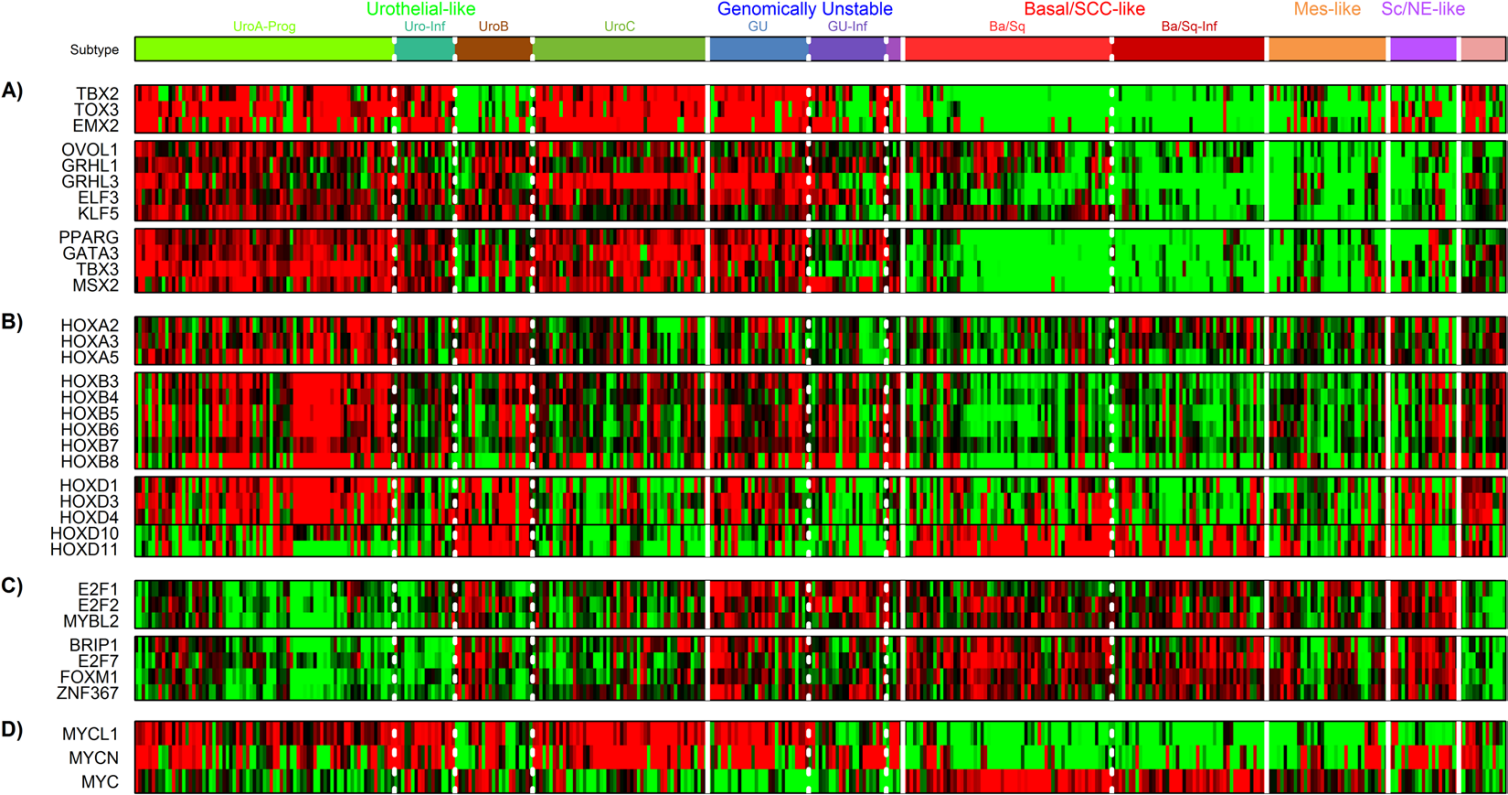
Transcription factor expression in the TCGA bladder cancer cohort. (**A)** Transcription factor gene clusters associated with the Uro and GU subtypes. (**B)** Expression of *HOXA*, *HOXB*, and *HOXD* families of transcription factors. Black horizontal line in the *HOXD* panel separate anterior and posterior *HOXD*-genes. (**C)** Transcription factor gene clusters associated with GU, Basal/SCC-like, Mes-like and Sc/NE-like subtypes. that include genes involved in late cell cycle regulation. (**D**) Expression of *MYC*-gene family members. Gene expression color codes; red, high expression; green, low expression.

We extracted 989 DNA binding transcription factors (TFs) from the TCGA gene expression data. These were subjected to a QTC analysis that produced 21 clusters encompassing 94 highly correlated TFs (Supplementary Fig. S5). To identify QTCs with similar expression across the samples, a hierarchical clustering using the medians of the QTCs was performed (Supplementary Fig. S6). This identified three major patterns of expression; one associated with infiltration of immune and stromal cells that included factors such as *FOXP3*, *RUNX3, TWIST1*, and *PRRX1*, a second associated with Uro subtypes and GU cases (Fig. 5A and B), and a third pattern associated with the GU, Basal/SCC-like, Mes-like, and Sc/NE subtypes (Fig. 5C). The group of factors primarily expressed by Uro and GU subtypes included *PPARG*, *GATA3*, *ELF3*, *TBX2*, and *TBX3*, all involved in differentiation of the urothelium (Fig. 5A). The same genes showed almost no expression in the Basal/SCC-like, Mes-like, and Sc/NE-like cases, indicating a fundamentally different state of differentiation in these subtypes. This difference was further emphasized by the expression of members of the *HOXA* and *HOXB* gene clusters (Fig. 5B), as well as *HOXD1*, -*D3*, and -*D4* genes. The posterior *HOXD10* and -*D11* genes were expressed in UroB as well as in Basal/SCC-like cases, reinforcing the similarity between the UroB and the Basal/SCC-like subtypes. In contrast, UroC was essentially negative for expression of all *HOXD*-genes. Transcription factors primarily expressed in GU, Basal/SCC-like, and Sc/NE-like cases included *FOXM1* and *MYBL2*, as well as *E2F1* and *E2F2* (Fig. 5C). FOXM1 and MYBL2 (B-MYB) interact with the MuvB complex to activate the late cycle by modulating E2F1 and E2F2 activities^19^. Their expression is indicative of the more aggressive growth patterns seen in GU, Basal/SCC-like, and particularly in the Sc/NE-subtype. In addition, we noticed a distinct expression pattern of the *MYC* family of transcription factors; *MYCL1*, *MYCN*, and *MYC* (Fig. 5D). *MYCL* was expressed by Uro and GU cases, *MYCN* by Uro and GU cases as well as by Sc/NE-like cases, whereas *MYC* expression was limited to the Basal/SCC-like cases. Taken together, the patterns of transcription factor expression expand the notion that the LundTax tumor cell phenotypes differ at their underlying gene regulatory systems^13^.

### LundTax classification of the TCGA cohort in relation to overall survival

**Figure 6:**
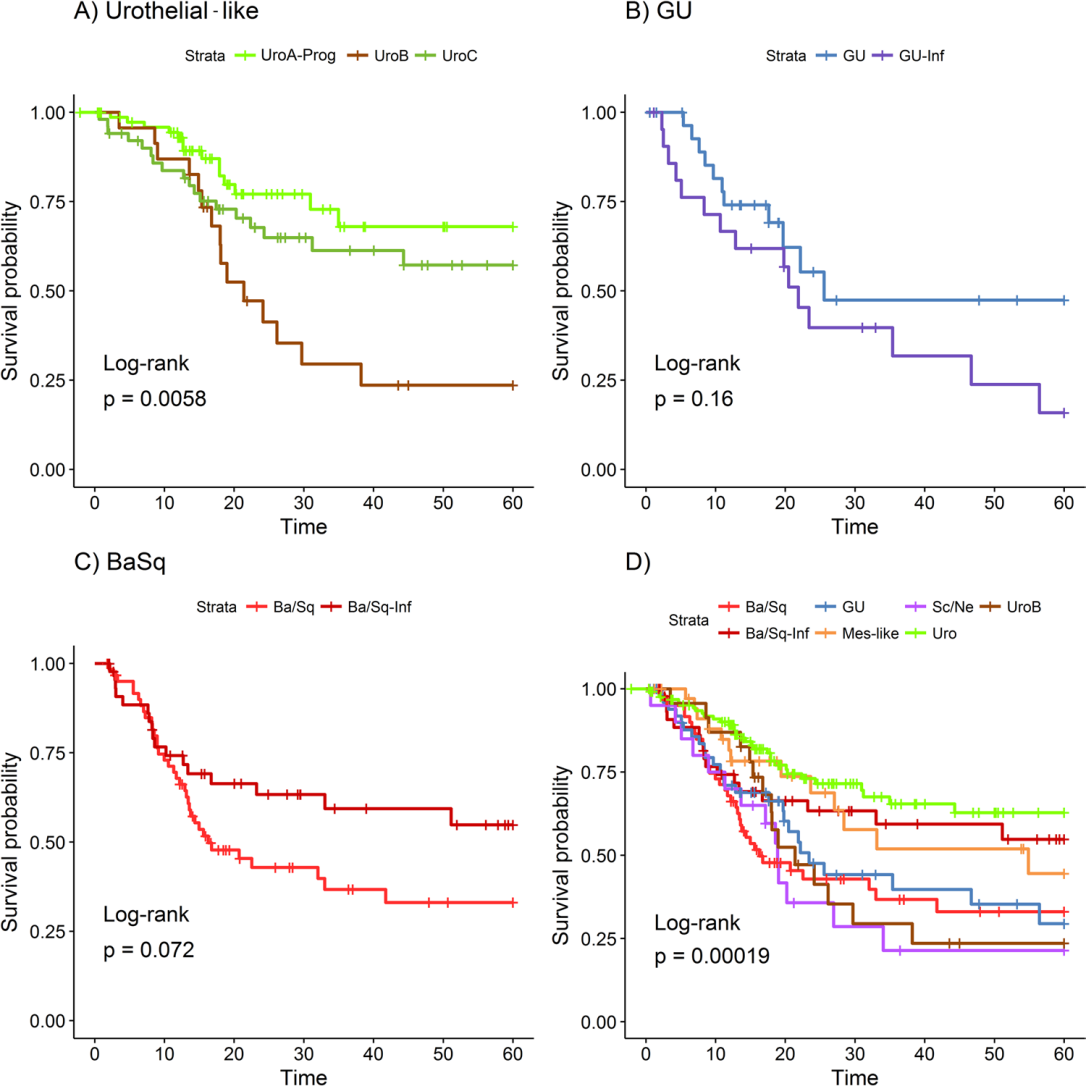
Kaplan-Meier curves for 5-year overall survival. TCGA bladder cancer cohort grouped by the molecular subtype based on LundTax classification. Groups with less than 20 samples (Uro-Inf, n = 18 and Inf, n = 14) were excluded from the analysis. (**A)** Five-year overall survival analysis for the Urothelial-like subgroups. (**B)** Five-year overall survival analysis for the Genomically Unstable subgroups. (**C)** Five-year overall survival analysis for the Basal/SCC-like subgroups. (**D)** Five-year overall survival analysis for the LundTax molecular subtypes. UroB was treated separately from the merged UroA-Prog/UroC group, GU and GU-Inf was merged into one GU group. Ba/Sq and Ba/Sq-Inf were treated as two separate entities.

We downloaded overall survival (OS) data for 407 patients in the TCGA data set. We then divided each major tumor cell phenotype into their respective subgroups i.e., Urothelial-like subtypes into UroA-Prog, UroB and UroC, Genomically Unstable into GU and GU-Inf, and Basal/SCC-like into Ba/Sq and Ba/Sq-Inf, and tested for within tumor cell phenotype differences. Among the Urothelial-like, UroB showed a significantly worse prognosis than UroA-Prog and UroC respectively (Fig. 6A) while UroA-Prog and UroC did not differ from each other (p = 0.18). No difference was observed in the GU vs. GU-Inf comparison (Fig. 6B), whereas a borderline significance was obtained for the Ba/Sq and Ba/Sq-Inf comparison (Fig. 6C). A Kaplan-Meier analysis was then performed with seven groups Uro (UroA-Prog and UroC merged), UroB, GU (GU and GU-Inf merged), Ba/Sq, Ba/Sq-Inf, Mes-like, and Sc/NE-like as separate entities (Fig. 6D). This analysis identified a bad prognosis group consisting of patients with UroB or Sc/NE-like tumors, with a survival probability of 0.22 at month 40, and a better prognosis group consisting of patients with Uro (UroA-Prog and UroC merged), Mes-like, or Ba/Sq-Inf tumors, with survival probability of 0.62 at month 40. The survival rate for the merged Uro group was compared with the rate for the merged GU group, both classified as “luminal” by the UNC and MDA classification systems, and the GU group was found to have a significantly worse OS (p = 0.0017) (Supplementary Fig. S7). Taken together, the LundTax classification identifies groups of patients with significantly different overall survival rates.

### UNC, MDA, and TCGA classification of the TCGA cohort in relation to LundTax classification

**Figure 7:**
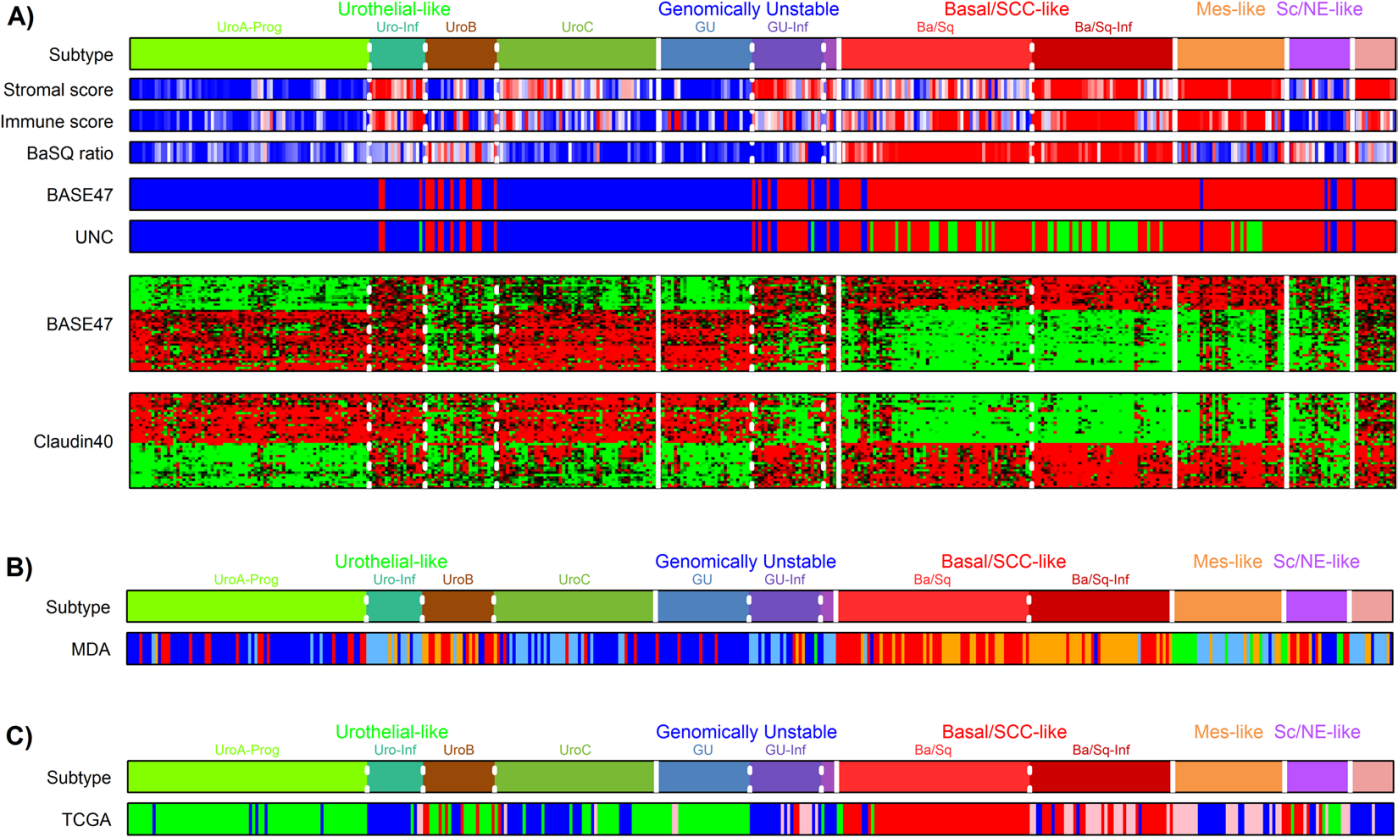
Comparison with UNC, MDA, and TCGA classification systems. (**A**) Comparison of the LundTax and UNC classification calls in the TCGA bladder cancer cohort. Top bar shows the LundTax classification of the TCGA samples. Stromal and immune score bars show the estimated scores for tumor purity based on the ESTIMATE tool^12^. Color codes; high score (red) indicates high-level infiltration; low score (blue) indicates low-level infiltration. Ba/Sq ratios were calculated for each sample as *KRT5* + *KRT14* − *FOXA1* − *GATA3* using log2 mRNA expression values. High ratio (red) indicate a Ba/Sq subtype, low ratio (blue) indicate non-Ba/Sq subtype. Bar indicated by “BASE47” shows classification using the BASE47 classifier^3^. Color codes, blue, Luminal; red, Basal. Bar indicated by “UNC” shows BASE47 classification with the Claudin-low 40 (BCL40)^4^ classification added. Color codes; blue, Luminal; red, Basal; green, Claudin-low. In the lower panels, heat maps of BASE47 and BCL40 signature genes are shown. (**B)** Comparison of the LundTax and MDA subtypes^5^. Color codes for the MDA classification; blue, Luminal; light blue, Luminal-TP53, red, Basal; orange, Basal-TP53; green, Double negative. (**C)** Comparison of the LundTax and TCGA I-IV subtypes^7^. Color codes for the TCGA classification; green, Cluster I; blue, Cluster II; red Cluster III; pink, Cluster IV. Vertical white lines separate major LundTax molecular subtypes; dotted lines, separate subgroups of subtypes.

We classified the TCGA samples according to the UNC classification system using the BASE47 classifier. Overall, tumors with Uro or GU subtypes were classified as “luminal”, and Basal/SCC-like, Mes-like, and Sc/NE-like as “basal” (Fig. 7A). We then applied the UNC Claudin 40 signature to the data. This signature appeared to mirror the BASE47 expression profile and in fact shows a strong correlation with this signature, r = 0.92, p < 10^−16^ for genes upregulated in the Basal subtype. The majority of the Claudin-low cases were of the Ba/Sq-Inf subtype i.e., infiltrated Basal/SCC-like cases (Fig. 7A). The five MDA classes Luminal, Luminal-P53, Basal, Basal-p53, and the small group of “double negative”, were then overlaid the LundTax grouping of the TCGA data (Fig. 7B). The Ba/Sq and the Ba/Sq-Inf groups coincided well with the MDA Basal and Basal-p53 respectively, whereas UroB was classified as Basal or Basal-p53 in almost all cases. The Uro and GU cases were classified as Luminal or its infiltrated counterpart, Luminal-p53, in almost all cases. However, very mixed classification results were obtained for the LundTax Mes-like and Sc/NE-like cases. The MDA “double negative” class was to a great majority included in the Mes-like class of tumors. We then classified the LundTax ordered cases according to the four-group TCGA principle (Fig. 7C). The TCGA Cluster I cases correspond well with Uro and GU cases with low infiltration, and Cluster II with Uro and GU cases infiltrated by immune and stromal cells. Cluster III cases correspond well with the less infiltrated Basal/SCC-like cases whereas Cluster IV matches with infiltrated Basal/SCC-like and Mes-like subtypes.

## Discussion

Recent reports indicate that the major non-surgical treatment modalities for advanced UC, systemic chemotherapy and immune-checkpoint inhibitors, may show different response rates between molecular subtypes^20,21^. Whether these associations will hold for clinical application or not, depends on our ability to stratify tumor samples into relevant molecular subtypes. A major approach to identify tumor molecular subtypes has been to perform genome wide gene expression followed by unsupervised hierarchical clustering. One often overlooked caveat of this approach is that advanced tumors, and consequently the clinically most challenging tumors, are infiltrated by non-tumorous cells. Even if care is taken to free biopsies from infiltrating cells, many tumors are networks of tumor and stromal/immunological cells. Under such circumstances it may be hard to arrive at statements on particular features associated with a given *tumor cell phenotype* i.e., the underlying tumor cells. To correct for this, we previously performed an extensive IHC based analysis using a large collection of tumor cell markers to characterize tumor cells proper^11^. By combining global gene expression and IHC analyses, we arrived at a refined classification system for urothelial carcinoma. In the present investigation, we applied a supervised classification approach to derive an mRNA-expression based classifier that identifies IHC defined *tumor cell phenotypes*, in both a low and high infiltration context. We applied this classifier to the independent but analogous TCGA set of tumors and made use of the extensive genomic information attached to the samples in this cohort to validate the biological relevance of the obtained classification.

We point to the large heterogeneity of the Urothelial-like group of tumors, previously named “Urobasal”^16^. This group was originally described in a non-muscle-invasive setting and was shown to include two variant forms, the UroA and the UroB. UroA and UroB share many molecular features but UroB also express markers typical for a basal-like cell type e.g., KRT5 and KRT14. We have previously shown that UroA tumors express anterior *HOXA* (*HOXA1*-*HOXA7*) as well as anterior *HOXB* (*HOXB1*-*HOXB8*) genes^22^. Anterior *HOXA* and *HOXB* expression are related to retinoid signaling and induced differentiation^23^, in line with the expression of *RXRA* and *PPARG* as well as *GATA3*, *FOXA1*, and *ELF3* in these tumors. Expression of the anterior *HOXA* and *HOXB* genes is maintained in the more advanced UroA, i.e., high risk T1 or ≥T2 tumors. The shared gene regulatory systems, as well as the shared genomic alterations, suggest that advanced UroA are progressed versions of the non-muscle invasive counterpart UroA, hence the name UroA-Prog. UroC, on the other hand, was first identified as a group of Urothelial-like tumors that cluster with Genomically Unstable tumors using global gene expression analysis^11^. However, UroC is distinct from GU by the expression of *FGFR3*, *CCND1*, and *RB1*, and by showing low expression of *CDKN2A*, features that UroC share with UroA, UroA-Prog and UroB tumors^14^. In addition, UroC shares several genomic alterations with the non-muscle invasive UroA, UroA-Prog, and UroB subtypes, such as losses of 9q, 11p, *CDKN2A*, and genomic amplifications of *CCND1*, indicating a strong genomic relationship. However, UroC was almost devoid of *FGFR3* mutations. This suggests that UroC have developed along a route independent of any hardwiring changes of this pathway making it possible to progress towards a GU-like phenotype, underscored by the frequent 2p25 (*YWHAQ*), 3p25 (*RAF1*), and 6p22 (*E2F3*/*CDKAL1*/*SOX4*) genomic amplifications seen in both UroC and in GU tumors. Even though the UroA-Prog, UroB and UroC share genomic and gene regulatory features, they differed with respect to patient overall survival, UroA-Prog and UroC tumors showed the best OS characteristics in the TCGA cohort, and patients with UroB showed the worst. We have previously noted the bad prognosis for patients with UroB tumors^10^. In contrast to Urothelial-like cases, Genomically Unstable tumors show frequent inactivation of *RB1* and high levels of *CDKN2A* (p16) expression. GU also showed the strongest enrichment for mutations in genes implicated in genomic instability, as well as the highest frequency of *TP53* mutations. This complies with the more complex genomic alterations seen in GU^14,16^. GU shares these features with Basal/SCC-like, Mes-like, and the Sc/NE-like subtypes. In addition, GU showed the largest non-silent mutational burden, making it a good candidate for immune checkpoint directed treatments^20,24^. Taken together, the Urothelial-like and Genomically Unstable subtypes, both classified as “luminal” by other systems, have distinct genomic properties and should be kept as separate entities^25^.

Tumors classified as Basal/SCC-like complied with the consensus definition, *KRT5*, *KRT14* high, and *FOXA1*, *GATA3* low, in almost all cases. Furthermore, the same group expressed *CDH3* (P-Cadherin) and *EGFR*, but not *ERBB2*/*ERBB3*, signifying a basal-like phenotype. We noticed a distinct antiparallel expression of the MYC family of transcription factors where *MYC* was almost exclusively expressed by the Basal/SCC-like subtype whereas *MYCL1* and *MYCN* were expressed in Urothelial-like and Genomically Unstable cases. The effects of the c-MYC oncoprotein can be pharmacologically reversed by targeting inhibitory BET bromodomain proteins, and one such compound (OTX015) has shown biological activity against triple-negative breast cancer^26^ as well as non-small cell lung cancer^27^. The specific overexpression of *MYC* in the Basal/SCC-like subtype combined with activity in its closest corresponding subtypes of breast- and lung cancer may warrant pre-clinical testing of this agent in Basal/SCC-like bladder cancer. The Mes-like subtype was distinct from both the Uro, GU and from the Basal/SCC-like subtypes by not expressing any of the class defining *KRT5*, *KRT14*, *FOXA1*, or *GATA3* genes, nor any of the class characteristic *EGFR* or *ERBB2* and *ERBB3* genes. In this sense, this subtype could be characterized as “multi negative”. On the other hand, the most characteristic feature of Mes-like tumor cells, tumor cell expression of *VIM* and *ZEB2*^11^, is not traceable using whole biopsy mRNA expression as Mes-like tumors are infiltrated by stromal cells. Irrespectively, the classifier readily identified a group of tumors as Mes-like.

The Sc/NE-like class of tumors is not well characterized at the molecular level. However, as the Mes-like subtype, Sc/NE-like cases express low levels of the class defining *KRT5*, *KRT14*, *FOXA1*, and *GATA3* genes, making it distinct from the Uro, GU, and Basal/SCC-like cases. Nevertheless, Sc/NE-like tumors are similar to GU tumors by showing frequent gains of 2p25 (*YWHAQ*), 3p25 (*RAF1*), and 6p22 (*E2F3*, *CDKAL1*, *SOX4*), as well as a strong activation of the FOXM1/MYBL2/E2F1/E2F2/MuvB driver of the cell cycle. In contrast to GU, the *MYC* gene family expression was limited to *MYCN*, a feature shared with neuroblastomas. In addition, Sc/NE-like tumors do not express any urothelial differentiation genes e.g., *UPKs*. This makes Sc/NE-like distinct from both Uro and GU as well as from Basal/SCC-like and Mes-like tumors. The robust identification of Sc/NE-like UC may be of unexpected clinical relevance; patients with small-cell or neuroendocrine histological variant tumors generally have a worse prognosis^28^ but respond well to “neuroendocrine” systemic chemotherapy regimens, e.g., the etoposide and ifosfamide containing IA-EP doublet^29^. It remains to be seen if the 5% of UC classified as Sc/NE-like according to our classifier would benefit similarly from a neuroendocrine treatment regime.

We argue that the underappreciated molecular complexity in invasive bladder cancer should be considered during clinical studies and trials. The molecular subtypes indicate that potentially targetable alterations, e.g., ERBB2 overexpression, may be found in different tumor subtypes^17,30^. Hence, it appears evident that the molecular context will be of importance when evaluating results from studies that explore therapeutic targeting of genomic alterations. This also applies to broader therapies, such as checkpoint inhibition, due to the associations between molecular subtypes and key features that could influence response, such as genomic instability, mutation load, and the degree and composition of the infiltrating immune and stromal cells. In our view, the ability to gain novel therapeutic insights increases substantially with a more comprehensive biological understanding of the tumor material.

We have shown that an mRNA classifier based on *tumor cell phenotypes* defined by extensive IHC analyses identifies groups of tumors with coherent mRNA gene expression, genomic alterations, and gene mutation patterns when applied to the independent TCGA cohort. We point to the importance of keeping the tumor cell phenotype approach apart from a global gene expression method. The latter does not separate signals from the tumor cells proper, from signals originating from infiltrating non-tumor cells. This blurs the analysis particularly as infiltration may vary among tumors of the same tumor cell phenotype. Highly infiltrated cases frequently converge to less well-defined “infiltrated” clusters of tumors. This leads to the possible necessity for a bi-nominal classification system consisting of the *tumor cell phenotype*, and its *context*; the context being the type and level of infiltrating non-tumor cells. Hence, to achieve a more accurate and complete description of bladder cancer specimens, we envision a system where tumor-cell phenotype is given, e.g., Basal/SCC-like, in combination with a score for immune- and stromal content, relieving the need for an “infiltrated” class of each subtype. Potentially, this scoring could be extended to include proportions of different types of immune cells^31^ or types of stromal cells. However, to accomplish this, mRNA subtype classifiers need to be trained on data sets that also contain tissue level phenotyping. The centroid-based classifier presented here represents the first iteration of such a next-generation subtype classifier.

## Material and Methods

### Gene expression data

For gene expression data (GEX), TCGA Bladder Cancer (BLCA) gene-level expression data (n = 426 samples, version 2016-08-16) was downloaded from UCSC Xena hub (http://xena.ucsc.edu) as log2 values of the normalized RSEM counts. This data was generated from level 3 TCGA GEX files with the suffix “.rsem.genes.normalized_results”. Only the tumor samples (n = 407) were kept. The dataset was median re-centered. Transcription factors genes (n = 989 genes) were extracted based on TFcheckpoint^32^, where only the human genes with evidence of specific DNA binding activity and regulator function of RNA polymerase II were included.

### Deriving an mRNA based classifier

The Sjödahl *et al*. 2017 cohort^11^ (GSE83586) was previously classified by IHC using antibodies for a large set of proteins into the Urothelial-like (Uro), including UroA-Prog, UroB, and UroC, the Genomically Unstable (GU), Basal/SCC-like, and the less frequent Mesenchymal-like (Mes-like), and Small-cell/Neuroendocrine-like (Sc/NE-like) subtypes, as well as their infiltrated counterparts. This approach firmly established the tumor cell phenotypes in each tumor independent of e.g., the presence of infiltrating non-tumor cells in the biopsies (Sjödahl *et al*. 2017). Global gene expression data for the same tumors was then used in a supervised fashion to build an mRNA centroid classifier that identifies the *IHC-defined* tumor classes. Gene feature selection was performed using the ClaNC R-package^33^, with “class” as the prior setting. Mean subtype expression of the selected genes were used as centroids. The reclassification accuracy was tested with increasing number of selected genes per tumor group (Supplementary Fig. S1). The final centroid was constructed using 100 genes per group where the classification recall rate plateaued and to provide redundancy when applying the centroid to other datasets. The centroids (Supplementary File S1) were derived using the mean expression of each subtype group for the 1200 selected genes.

### Mutation analysis

The mutation annotation format (MAF) file for TCGA bladder cancer cohort (n = 395 samples, version 2016-01-28) was downloaded from Broad Institute of MIT and Harvard (10.7908/C1MW2GGF). This MAF file shares the gene names used in the MutSigCV tool (version 1.41)^34^. Only samples with both mutation and GEX data were kept for the downstream analyses (n = 389). For association with the molecular subtypes, only genes with mutation frequencies >5% (n = 351 genes) were tested by Fisher’s exact test. Bonferroni correction was made to correct for multiple testing. The MutSigCV tool was used to find significantly mutated genes (n = 30 genes, false discovery rate (q-value) <0.05). The gene ontology (GO) terms from “GO_Biological_Process” column in the MAF file were used to group gene mutations that affect the same biological process. A biological process was considered altered when any gene associated with the process is mutated. Biological process that altered in >3% of cases were selected, and Fisher’s exact test was then performed to find any association of altered processes with the molecular subtypes.

### Copy number data processing

Segmented copy number data (Level 3, Affymetrix SNP Array 6.0 data without germline copy number variants) for fresh frozen TCGA bladder cancer cohort (n = 410 samples, version 2016-01-28) was downloaded from Broad Institute of MIT and Harvard. Only tumor samples (n = 405 samples) were kept, and 400 samples of the cohort had GEX data. An automatic baseline estimation step was performed using CopyNumber450kCancer R package^35^. Briefly, in each sample, the highest peak in the density function of the segments was selected to be the baseline in that sample. Then the copy number plots were manually revised to check the baseline position and to determine the cutoffs for gains, deletions, amplification, and homozygous deletions in each sample. Estimated tumor purity from Yoshihara *et al*.^36^ and the density function of the segments were used to assist the determination of the cutoffs. Used cutoffs for the samples are given in the Supplementary Table S3. The frequently altered regions in the cohort were determined using the R packages CNTools^37^ and cghMCR^38^. Losses seen in more than 20%, and gains in more than 25% of the samples were included for further analyses (Supplementary Fig. S8 and Supplementary Table S4). After determining the frequently altered regions, samples were analyzed for the presence of aberrations using the following criteria; (1) for individual genes 100% should be altered for gains, and >50% for deletions, (2) for regions with size <5 Mbp, >75% should be altered for both gains and deletions, and (3) for regions with size > 5Mbp and chromosomal arms, >50% should be altered for both gains and deletions. The frequent altered regions were tested for association with the molecular subtypes by Fisher’s exact test.

### Bladder cancer classification systems

TCGA bladder cancer samples with GEX data was, in addition to the LundTax classification system, classified according to three additional classification systems; the University of North Carolina (UNC) classification system using the BASE47 and Claudin-low 40 (BCL40) classifiers^3,4^, the MD Anderson (MDA) Cancer Center classification system^5^, and The Cancer Genome Atlas (TCGA) classification system^7^.

### Statistical analysis

Statistical tests were performed using the R environment. Fisher’s exact test was used for testing the association with the molecular subtypes. Bonferroni correction was used for multiple test correction when needed. The significance threshold was 0.05 for all the statistical tests. The highly-infiltrated subtype (Inf) was excluded from Fisher’s exact tests. To find TFs with similar expression patterns, Quality Threshold (QT) clustering^39^ was performed. The used threshold distance was 0.4 and the size of the cluster was at least 3 genes. Only QT clusters with expression variance >1 were kept, resulting in 21 QT clusters containing 94 TF genes. Clinical data for TCGA bladder cancer cohort (n = 407) was downloaded from Broad Institute of MIT and Harvard. (version 2016/01/28; http://firebrowse.org/?cohort=BLCA). Kaplan-Meier analysis and log-rank test were used for 5-year overall survival analysis. Samples grouped by their molecular subtype based on LundTax classification. Groups with less than 20 samples (i.e. Uro-Inf, n = 18 and Inf, n = 14) were excluded from the analysis.

### Data availability

All datasets used in the current study are publically available as described in the methods section.

## Electronic supplementary material

Supplementary information

Supplementary File 1
