# Supplementary material for: A validation and extended description of the Lund taxonomy for urothelial carcinoma using the TCGA cohort
Source: Sci Rep. 2018 Feb 27;8:3737. doi: 10.1038/s41598-018-22126-x (PMC5829240; doi:10.1038/s41598-018-22126-x)
Supplement: Supplementary file 1 — Supplementary information [file 41598_2018_22126_MOESM1_ESM.pdf]

Supplementary

A validation and extended description of the Lund taxonomy for urothelial carcinoma using the TCGA cohort.

Authors: Nour-al-dain Marzouka, Pontus Eriksson, Carlos Rovira, Fredrik Liedberg, Gottfrid Sjödahl, Mattias Höglund

Supplementary Figures

Supplementary Figure S1. Classifier accuracy. The "phenotype-informed" twelve-group split of the Sjödahl 2017 cohort[1] was used to build a phenotype-informed mRNA centroid classifier. Gene selection was performed using the ClaNC R-package[2]. The reclassification accuracy was tested with increasing number of selected genes per tumor group. The centroid was constructed using 100 genes per group where the classification recall rate plateaued and to provide redundancy when applying the centroids to other datasets. The centroids were constructed using the mean expression of each subtype group for the 1200 selected genes.

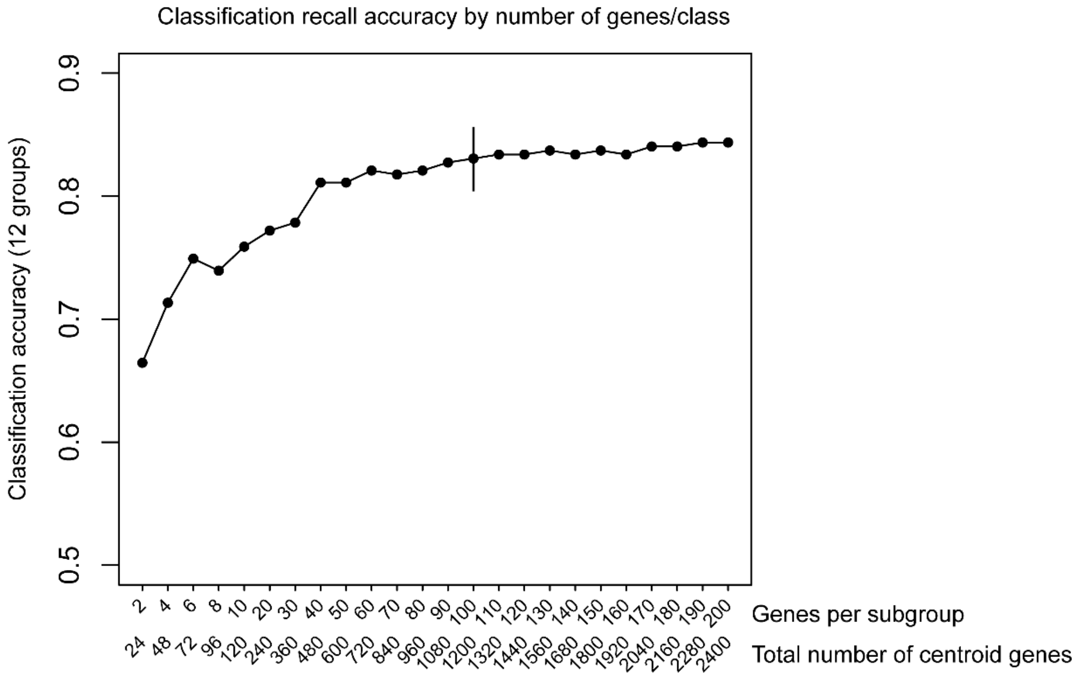

Supplementary Figure S2. Significantly mutated genes. Plot showing significantly mutated genes identified by MutSigCV (false discovery rate <0.05). Genes are sorted based on the mutation frequency in the whole cohort. Gray: unavailable information. Vertical lines. Red lines, separate major molecular subtypes; dotted lines, separate subgroups of subtypes. Uro: Urothelial-like; GU: Genomically Unstable; Basal/SCC-like: Basal/Squamous Cell Carcinoma like; Mes-like: Mesenchymal-like; Sc/NE-like: Small cell/Neuroendocrine-like; Ba/Sq: Basal-Squamous-like; Inf: Infiltrated.

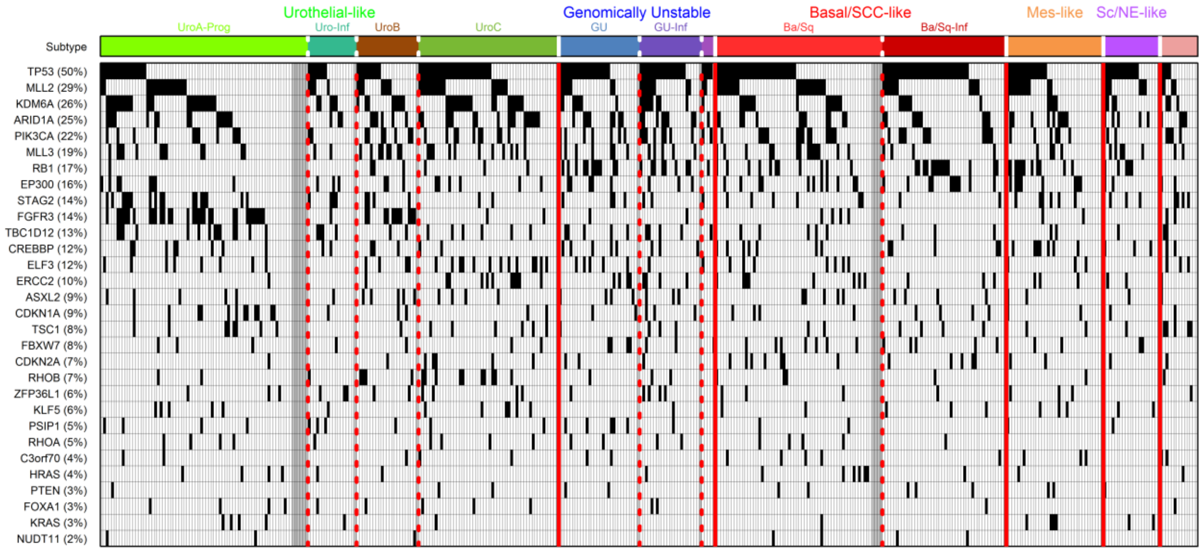

Supplementary Figure S3. Copy number alteration profiles for bladder cancer molecular subtypes in TCGA cohort (n = 400). Genomic regions gained in >100 samples were considered as frequent gains, Genomic regions lost in >80 samples were considered as frequent losses. The frequent genomic alterations proportions were compared among the molecular subtypes using Fisher's exact test. This figure shows the significant ($p<0.05$) alterations **A**) with multiple test correction (Bonferroni correction) and **B**) without multiple test correction. In (**A**) samples were sorted according to the gene expression data clustering, while in (**B**) the samples were sorted according to the alteration frequency in the complete cohort. Gene names in parentheses indicate that the gene coordinates were used to report this region (see Supplementary Table S3 for details). Heat map. Red: gain; dark red: amplification; blue: loss; dark blue: homozygous deletion; white: no alteration; gray: unavailable information. Vertical lines. Black lines, separate major molecular subtypes; dotted lines, separate subgroups of subtypes. Uro: Urothelial-like; GU: Genomically Unstable; Basal/SCC-like: Basal/Squamous Cell Carcinoma like; Mes-like: Mesenchymal-like; Sc/NE-like: Small cell/Neuroendocrine-like; Ba/Sq: Basal-Squamous-like; Inf: Infiltrated.

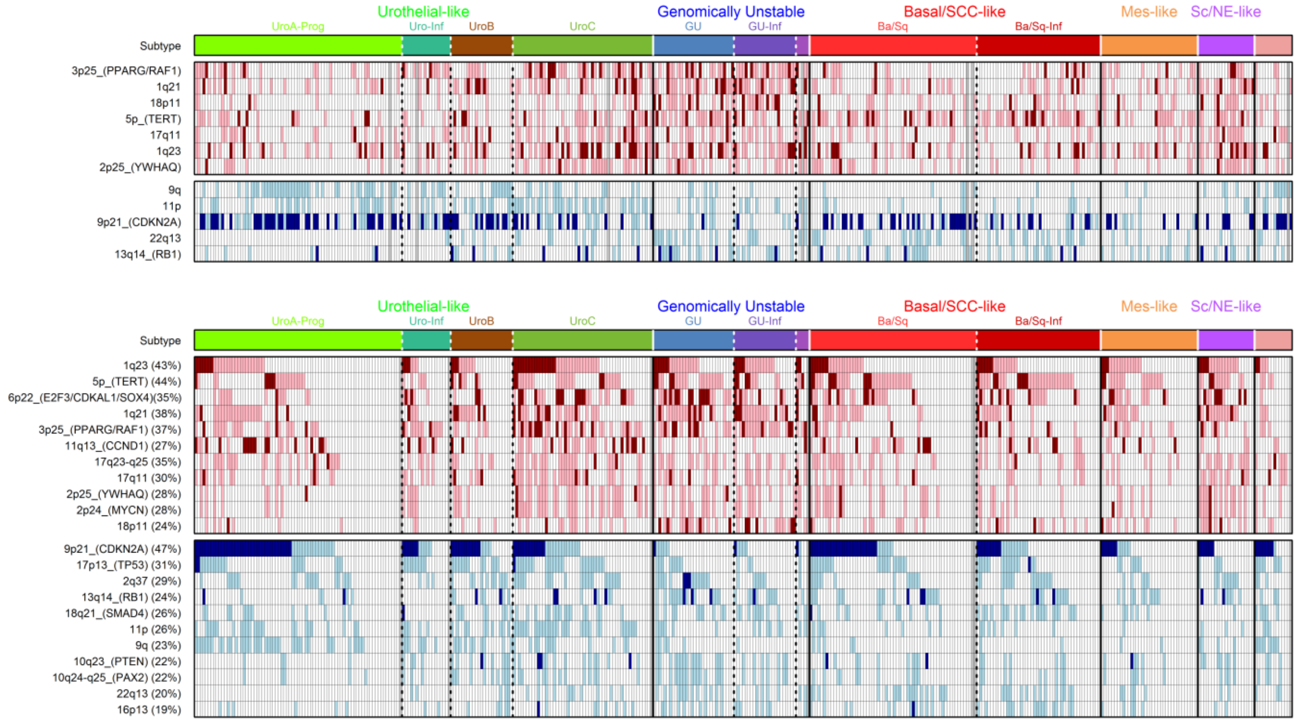

Supplementary Figure S4. Gene expression by copy number alterations. A) The 2p25 amplicon is frequently amplified in UroC and GU cases and includes the *YWHAQ* gene that shows strong correlated expression with amplification. **B** & **C)** The 3p25 region is frequently amplified in UroC and GU cases and contains two closely linked genes *PPARG* and *RAF1*. Both *PPARG* and *RAF1* showed increased expression when amplified, however, whereas *RAF1* expression was strictly associated with gene copy changes *PPARG* expression was not. Notably, several of the cases with amplified *PPARG* genes in fact showed downregulation of the gene. **D)** Strong association between 11q13 (*CCND1*) copy number and *CCND1* expression.

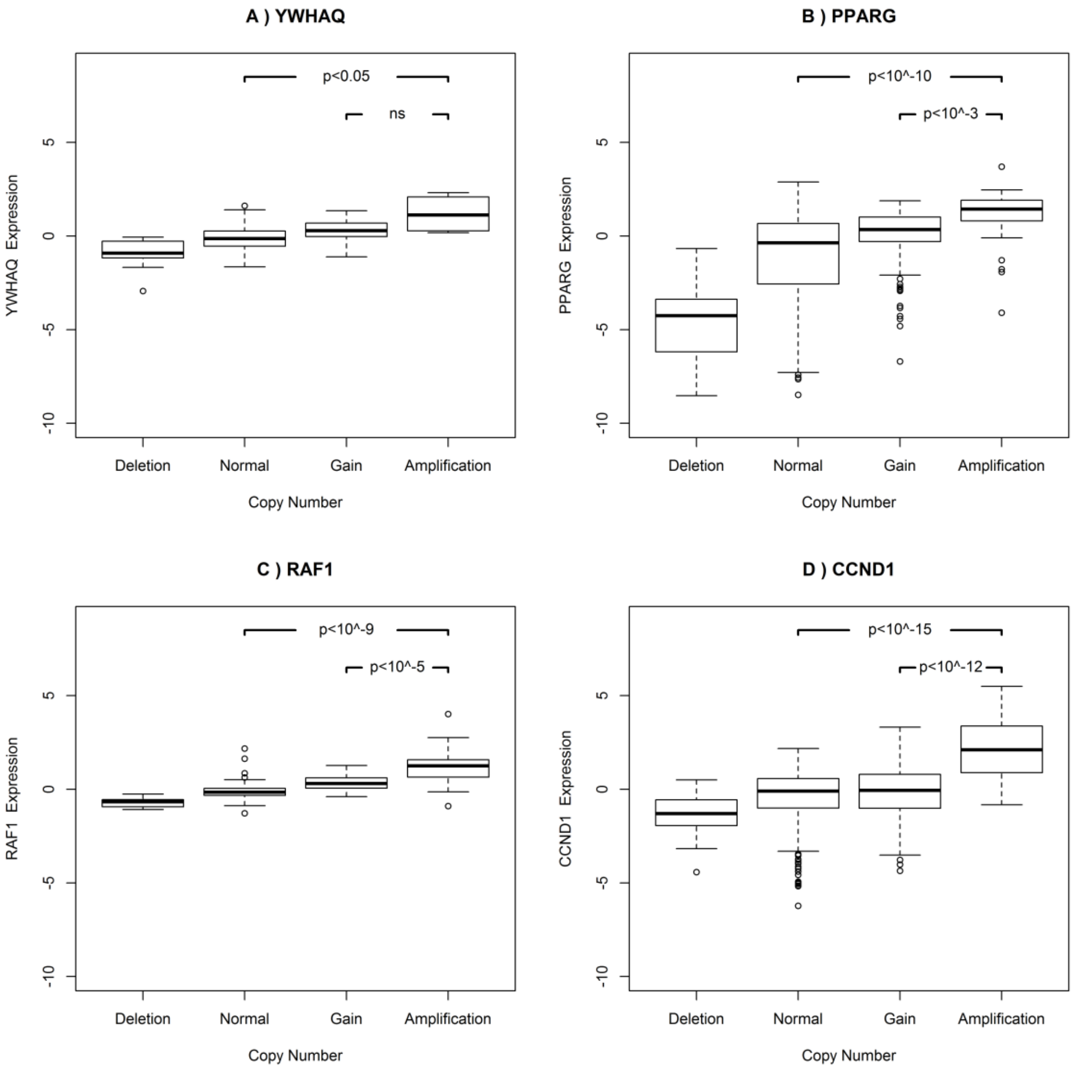

Supplementary Figure S5. Transcription factor clusters. Transcription factors (TFs) with similar expression patterns were clustered by quality threshold clustering[3]. The used threshold distance was 0.4 and the size of the cluster was at least 3 genes. Only QT clusters with expression variance more than 1 were kept, resulting in 21 QT clusters containing 94 TF genes. Uro: Urothelial-like; GU: Genomically Unstable; Basal/SCC-like: Basal/Squamous Cell Carcinoma like; Mes-like: Mesenchymal-like; Sc/NE-like: Small cell/Neuroendocrine-like; Ba/Sq: Basal-Squamous-like; Inf: Infiltrated.

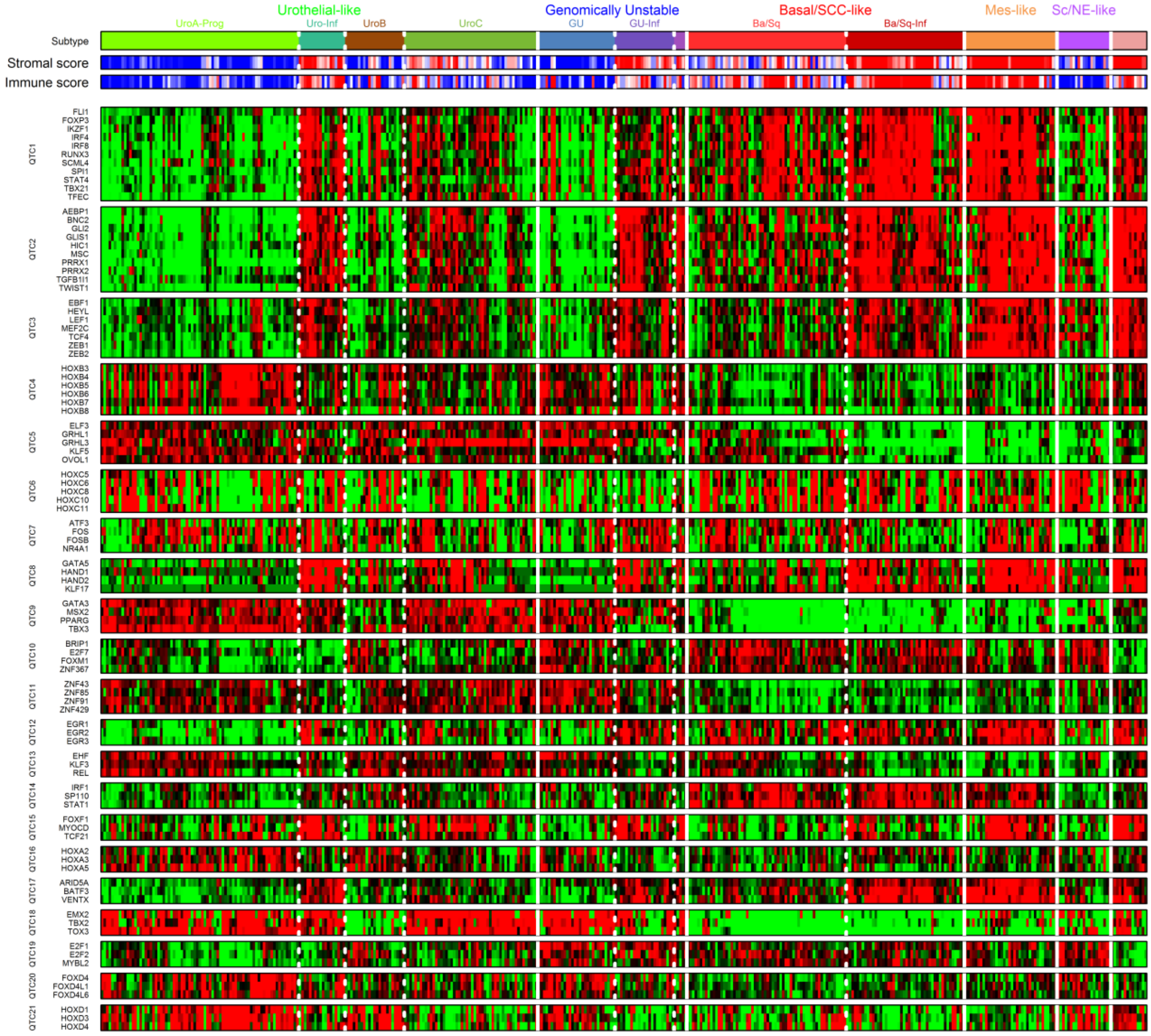

Supplementary Figure S6. Clustering of transcription factor QTCs. TF QTCs (21 clusters) were hierarchically clustered using the median gene expression of each QTC. The QTCs clustered in 4 groups A, B, C, and D.

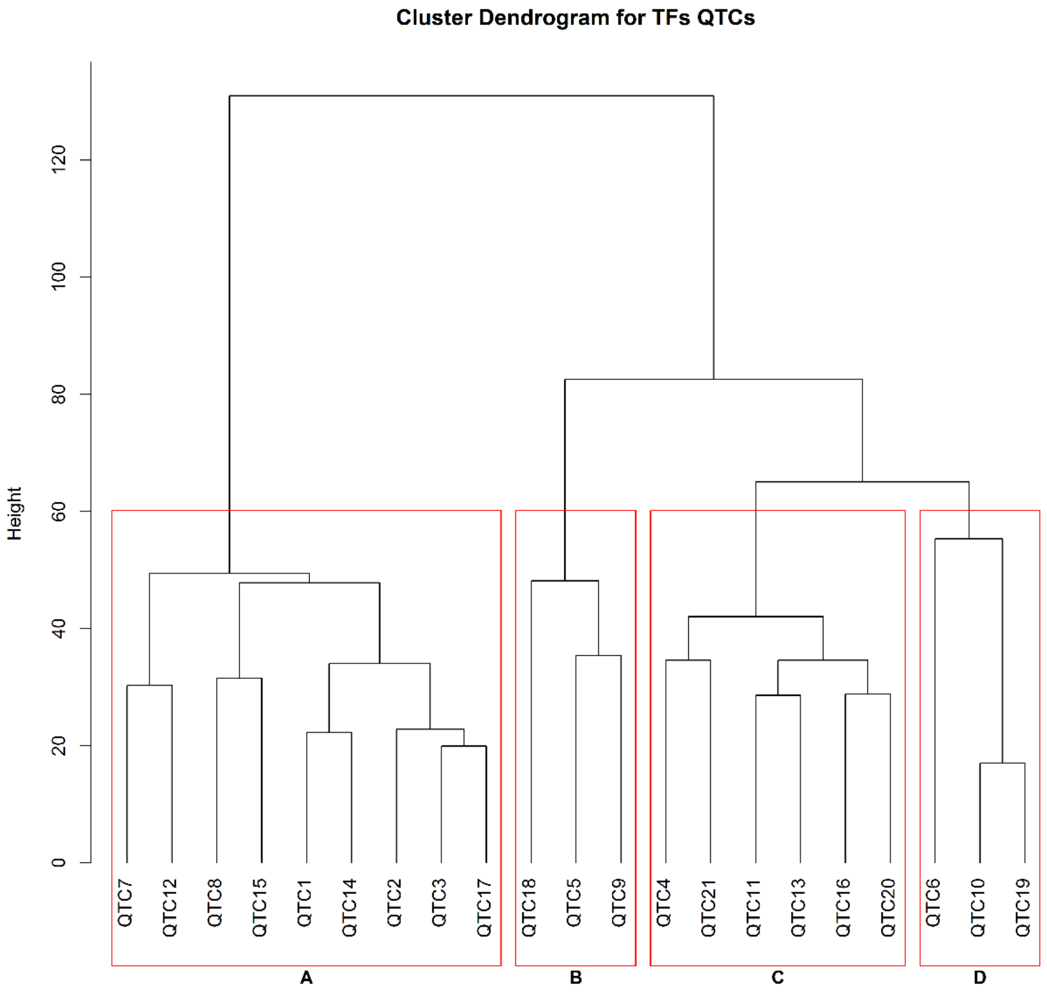

Supplementary Figure S7. Kaplan-Meier plot for 5-year overall survival of UroA-Prog and UroC samples versus Genomically Unstable samples. Significant difference in overall 5-years survival was shown between the two groups in the TCGA bladder cancer cohort (Log-rank, p=0.0017). Groups with less than 20 samples (i.e. Uro-Inf, n=18; GU-Inf-2, n=5) were excluded from the analysis. GU: Genomically Unstable.

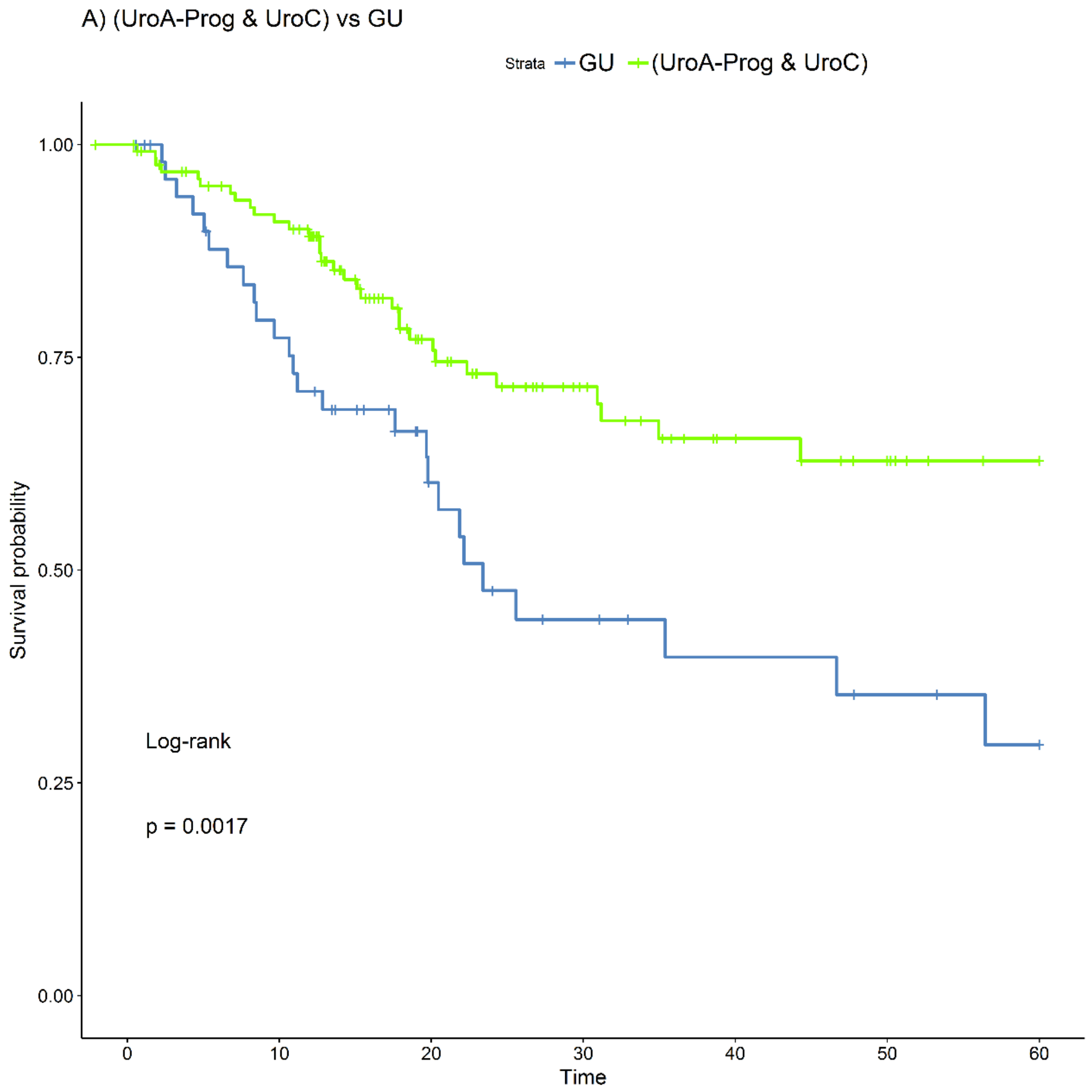

Supplementary Figure S8. Copy number frequency in the TCGA bladder cancer cohort. Frequency plot of genomic alterations in the TCGA cohort (n=405). X axis indicate chromosomes. Y axis indicates the number of samples with gains and losses. The horizontal lines indicate the thresholds for frequent gains (100 samples) and frequent deletions (80 samples). Green line represents gains, while the red line represents the deletions.

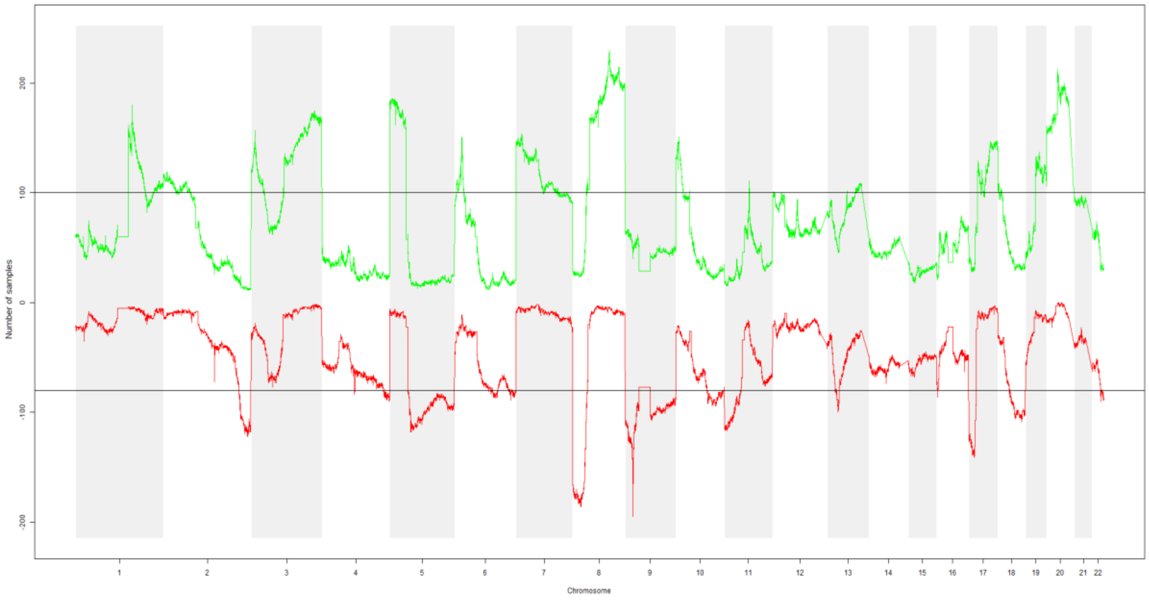

Supplementary Tables

Supplementary Table S1. Reclassification of the Sjödahl 2017 dataset[1] using centroids based on tumor cell phenotype information.

**Subtype Class***	Urothelial-like 116/133 (87%)				Genomically Unstable 59/66 (89%)			Basal/SCC-like 60/62 (97%)		Mes-like 16/16 (100%)	Sc/NE-like 24/24 (100%)	Infiltrated*** 4/6 (68%)
**Subclass Clusters ****	UroA-Prog 32/41 (78%)	Uro Inf 21/33 (64%)	UroB 17/24 (71%)	UroC 30/35 (85%)	GU 28/31 (90%)	GU Inf 2 17/23 (74%)	GU Inf 1 10/12 (83%)	Ba/Sq 21/22 (96%)	Ba/Sq Inf 35/40 (88%)	Mes-like 16/16 (100%)	Sc/NE-like 24/24 (100%)	Infiltrated 4/6 (68%)

*) Class accuracy: 279/307 (91%).

**) Subclass accuracy: 255/307 (83%).

***) Highly infiltrated tumors with uncertain class.

Uro: Urothelial-like; GU: Genomically Unstable; Basal/SCC-like: Basal/Squamous Cell Carcinoma like; Mes-like: Mesenchymal-like; Sc/NE-like: Small cell/Neuroendocrine-like; Ba/Sq: Basal-Squamous-like; Inf: Infiltrated.

Supplementary Table S2. Differentially mutated biological processes GO terms among the molecular subtypes in TCGA bladder cancer cohort (n=389). Mutated genes were grouped based on their biological processes GO terms, biological process that altered in >3% of cases were selected and Fisher's exact test was performed to find association with the molecular subtypes in LundTax classification system. The GO terms with p<0.05 (Bonferroni corrected) are shown here.

Gene Ontology (GO) terms for Biological Processes (BP)	GO number	p.value	p.value (Bonferroni)
sister chromatid biorientation	(GO:0031134)	2.75E-10	1.468E-06
regulation of lipid kinase activity	(GO:0043550)	5.02E-10	2.68238E-06
neuron apoptotic process	(GO:0051402)	9.79E-10	5.23313E-06
glial cell apoptotic process	(GO:0034349)	1.29E-09	6.88565E-06
bone maturation	(GO:0070977)	1.69E-09	9.05299E-06
cell morphogenesis involved in neuron differentiation	(GO:0048667)	2.25E-09	1.20294E-05
alveolar secondary septum development	(GO:0061144)	2.28E-09	1.21881E-05
regulation of centromere complex assembly	(GO:0090230)	2.48E-09	1.32615E-05
fibroblast growth factor receptor apoptotic signaling pathway	(GO:1902178)	3.82E-09	2.04276E-05
negative regulation of developmental growth	(GO:0048640)	3.82E-09	2.04276E-05
positive regulation of mitotic metaphase/anaphase transition	(GO:0045842)	6.22E-09	3.32317E-05
enucleate erythrocyte differentiation	(GO:0043353)	1.83E-08	9.80878E-05
epithelial cell fate commitment	(GO:0072148)	1.99E-08	0.00010642
central nervous system myelination	(GO:0022010)	4.31E-08	0.000230551
protein localization to chromosome, centromeric region	(GO:0071459)	4.63E-08	0.000247651
cellular response to glucose starvation	(GO:0042149)	5.81E-08	0.000310547
maintenance of mitotic sister chromatid cohesion	(GO:0034088)	1.46E-07	0.000782404
negative regulation of transcription involved in G1/S transition of mitotic cell cycle	(GO:0071930)	1.85E-07	0.000990199
intrinsic apoptotic signaling pathway by p53 class mediator	(GO:0072332)	1.91E-07	0.001020532
myoblast differentiation	(GO:0045445)	2.03E-07	0.001084273
hepatocyte apoptotic process	(GO:0097284)	2.92E-07	0.001561043
negative regulation of transcription from RNA polymerase II promoter during mitosis	(GO:0007070)	3.18E-07	0.001697498
protein localization	(GO:0008104)	5.72E-07	0.003056545
negative regulation of neuroblast proliferation	(GO:0007406)	7.96E-07	0.004256669
endochondral bone growth	(GO:0003416)	8.68E-07	0.00464002
regulation of cohesin localization to chromatin	(GO:0071922)	1.24E-06	0.006626703
negative regulation of G1/S transition of mitotic cell cycle	(GO:2000134)	1.72E-06	0.009172022
oligodendrocyte apoptotic process	(GO:0097252)	2.51E-06	0.013416862
base-excision repair	(GO:0006284)	2.56E-06	0.013670853
axonogenesis involved in innervation	(GO:0060385)	2.75E-06	0.014704737
chondrocyte proliferation	(GO:0035988)	2.85E-06	0.015225675
positive regulation of cell cycle arrest	(GO:0071158)	2.87E-06	0.015318529
embryonic organ development	(GO:0048568)	2.89E-06	0.015437881
response to antibiotic	(GO:0046677)	3.31E-06	0.017715805
neuron projection development	(GO:0031175)	3.39E-06	0.018102084
negative regulation of transforming growth factor beta receptor signaling pathway	(GO:0030512)	3.43E-06	0.018349452
positive regulation of MAPKKK cascade by fibroblast growth factor receptor signaling pathway	(GO:0090080)	3.63E-06	0.019389922
positive regulation of protein oligomerization	(GO:0032461)	4.02E-06	0.021515629
regulation of mitochondrial membrane permeability involved in apoptotic process	(GO:1902108)	4.33E-06	0.023169265
rRNA transcription	(GO:0009303)	4.73E-06	0.025287282
negative regulation of mitosis	(GO:0045839)	5.20E-06	0.027806837
positive regulation of cardiac muscle cell apoptotic process	(GO:0010666)	5.26E-06	0.02812912
positive regulation of histone deacetylation	(GO:0031065)	5.37E-06	0.028708021
positive regulation of cell aging	(GO:0090343)	5.45E-06	0.029139582
nucleotide-excision repair	(GO:0006289)	6.38E-06	0.034116452
negative regulation of helicase activity	(GO:0051097)	6.41E-06	0.034271276
ER overload response	(GO:0006983)	6.58E-06	0.03516673
cell cycle arrest	(GO:0007050)	6.74E-06	0.036021093
DNA damage response, signal transduction by p53 class mediator	(GO:0030330)	6.95E-06	0.037133001
cell aging	(GO:0007569)	9.22E-06	0.049314239

Supplementary Table S3. Adaptive cutoffs for gains, deletions, amplification, and homozygous deletions in each sample in bladder cancer TCGA cohort (n=405).

TCGA Bladder Cancer ID	Amplification cutoff	Gain cutoff	Deletion cutoff	Homozygous deletion cutoff
TCGA-2F-A9KO-01A-11D-A38F-01	0.303	-0.047	-0.317	-0.717
TCGA-2F-A9KP-01A-11D-A38F-01	0.468	0.148	-0.512	-0.942
TCGA-2F-A9KQ-01A-11D-A38F-01	1.114	0.364	-0.346	-0.976
TCGA-2F-A9KR-01A-11D-A38F-01	0.738	0.518	-0.382	-0.832
TCGA-2F-A9KT-01A-11D-A38F-01	0.739	0.379	-0.291	-0.661
TCGA-2F-A9KW-01A-11D-A38F-01	0.738	0.418	-0.332	-0.772
TCGA-4Z-AA7M-01A-11D-A390-01	0.481	0.071	-0.239	-0.709
TCGA-4Z-AA7N-01A-11D-A390-01	0.406	0.035	-0.038	-0.512
TCGA-4Z-AA7O-01A-31D-A390-01	0.640	0.317	-0.345	-0.918
TCGA-4Z-AA7Q-01A-11D-A390-01	0.360	0.134	-0.059	-0.310
TCGA-4Z-AA7R-01A-11D-A390-01	0.785	0.164	-0.393	-0.765
TCGA-4Z-AA7S-01A-11D-A390-01	0.710	0.193	-0.549	-0.977
TCGA-4Z-AA7W-01A-11D-A390-01	0.529	0.190	-0.294	-0.545
TCGA-4Z-AA7Y-01A-11D-A390-01	1.045	0.504	-0.522	-1.038
TCGA-4Z-AA80-01A-11D-A390-01	0.938	0.631	-0.540	-0.854
TCGA-4Z-AA81-01A-11D-A390-01	0.498	0.070	-0.261	-0.673
TCGA-4Z-AA82-01A-11D-A390-01	0.530	0.174	-0.415	-0.738
TCGA-4Z-AA83-01A-11D-A390-01	0.915	0.286	-0.465	-1.014
TCGA-4Z-AA84-01A-11D-A390-01	0.650	0.303	-0.424	-1.118
TCGA-4Z-AA86-01A-11D-A390-01	0.580	0.095	-0.074	-0.518
TCGA-4Z-AA87-01A-11D-A390-01	0.502	-0.071	-0.394	-0.765
TCGA-4Z-AA89-01A-11D-A390-01	0.702	0.250	-0.170	-0.824
TCGA-5N-A9KI-01A-31D-A42D-01	0.845	0.304	-0.302	-0.738
TCGA-5N-A9KM-01A-11D-A42D-01	0.626	0.133	-0.424	-0.836
TCGA-BL-A0C8-01A-11D-A273-01	0.486	0.050	-0.297	-0.758
TCGA-BL-A13I-01A-11D-A13V-01	0.486	0.074	-0.200	-0.491
TCGA-BL-A13J-01A-11D-A273-01	0.477	-0.048	-0.734	-1.162
TCGA-BL-A3JM-01A-12D-A219-01	0.673	-0.062	-0.498	-0.958
TCGA-BL-A5ZZ-01A-31D-A30D-01	0.471	0.003	-0.199	-0.546
TCGA-BT-A0S7-01A-11D-A10T-01	0.596	-0.009	-0.130	-0.477
TCGA-BT-A0YX-01A-11D-A10T-01	0.730	0.133	-0.271	-0.618
TCGA-BT-A20J-01A-11D-A14V-01	0.454	0.090	-0.443	-0.782
TCGA-BT-A20N-01A-11D-A14V-01	0.878	0.183	-0.455	-0.810
TCGA-BT-A20O-01A-21D-A14V-01	0.478	0.050	-0.160	-0.451
TCGA-BT-A20P-01A-11D-A14V-01	0.779	0.117	-0.465	-1.086
TCGA-BT-A20Q-01A-11D-A14V-01	0.705	0.229	-0.345	-0.902
TCGA-BT-A20R-01A-12D-A16M-01	0.298	0.088	-0.146	-0.404
TCGA-BT-A20T-01A-11D-A14V-01	0.806	0.297	-0.405	-0.930

TCGA-BT-A20U-01A-11D-A14V-01	0.492	0.250	-0.138	-0.275
TCGA-BT-A20V-01A-11D-A14V-01	0.367	-0.263	-0.820	-1.378
TCGA-BT-A20W-01A-21D-A14V-01	0.819	0.133	-0.231	-0.852
TCGA-BT-A20X-01A-11D-A16M-01	0.421	0.090	-0.201	-0.491
TCGA-BT-A2LA-01A-11D-A18E-01	0.601	0.060	-0.336	-0.748
TCGA-BT-A2LB-01A-11D-A18E-01	0.579	0.111	-0.115	-0.446
TCGA-BT-A2LD-01A-12D-A20B-01	0.490	0.062	-0.318	-0.544
TCGA-BT-A3PH-01A-11D-A21Y-01	0.790	0.225	-0.461	-0.905
TCGA-BT-A3PJ-01A-21D-A21Y-01	0.423	0.020	-0.319	-0.651
TCGA-BT-A3PK-01A-21D-A21Y-01	0.262	0.012	-0.101	-0.287
TCGA-BT-A42B-01A-32D-A23L-01	0.717	0.386	-0.268	-0.752
TCGA-BT-A42C-01A-11D-A23L-01	0.751	0.274	-0.686	-0.904
TCGA-BT-A42E-01A-11D-A23T-01	0.456	0.093	-0.182	-0.973
TCGA-BT-A42F-01A-11D-A23T-01	0.198	0.028	-0.117	-0.327
TCGA-C4-A0EZ-01A-21D-A10T-01	0.554	0.206	-0.472	-0.787
TCGA-C4-A0F0-01A-12D-A10T-01	0.787	0.230	-0.465	-0.820
TCGA-C4-A0F6-01A-11D-A10T-01	0.462	0.050	-0.184	-0.571
TCGA-CF-A1HR-01A-11D-A13V-01	0.760	0.130	-0.338	-0.742
TCGA-CF-A1HS-01A-11D-A13V-01	0.438	-0.079	-0.466	-0.797
TCGA-CF-A27C-01A-11D-A16M-01	0.736	0.187	-0.345	-0.983
TCGA-CF-A3MF-01A-12D-A219-01	0.785	0.099	-0.297	-0.765
TCGA-CF-A3MG-01A-11D-A20B-01	0.856	0.444	-0.331	-0.735
TCGA-CF-A3MH-01A-11D-A20B-01	0.972	0.334	-0.368	-0.990
TCGA-CF-A3MI-01A-11D-A20B-01	0.644	0.272	-0.398	-0.963
TCGA-CF-A47S-01A-11D-A23T-01	0.575	0.122	-0.386	-0.854
TCGA-CF-A47T-01A-11D-A23T-01	0.764	0.280	-0.455	-0.996
TCGA-CF-A47V-01A-11D-A23T-01	0.718	0.226	-0.461	-1.082
TCGA-CF-A47W-01A-11D-A23T-01	0.846	0.168	-0.349	-0.801
TCGA-CF-A47X-01A-31D-A23T-01	0.546	-0.100	-0.512	-1.037
TCGA-CF-A47Y-01A-11D-A23T-01	0.726	0.185	-0.380	-0.832
TCGA-CF-A5U8-01A-11D-A288-01	0.750	0.370	-0.615	-0.970
TCGA-CF-A5UA-01A-11D-A288-01	0.962	0.130	-0.403	-1.145
TCGA-CF-A7I0-01A-22D-A34T-01	0.881	0.267	-0.201	-0.807
TCGA-CF-A8HX-01A-11D-A363-01	0.940	0.327	-0.610	-1.005
TCGA-CF-A8HY-01A-11D-A363-01	0.646	0.105	-0.259	-0.840
TCGA-CF-A9FF-01A-11D-A38F-01	0.856	0.170	-0.290	-0.896
TCGA-CF-A9FH-01A-11D-A38F-01	0.861	0.223	-0.383	-1.028
TCGA-CF-A9FL-01A-11D-A38F-01	0.691	0.158	-0.666	-1.013
TCGA-CF-A9FM-01A-11D-A38F-01	0.666	0.230	-0.456	-1.045
TCGA-CU-A0YN-01A-21D-A10T-01	0.435	0.039	-0.131	-0.518
TCGA-CU-A0YO-01A-11D-A10T-01	0.235	0.050	-0.063	-0.306
TCGA-CU-A0YR-01A-12D-A10T-01	0.700	0.070	-0.245	-0.535
TCGA-CU-A3KJ-01A-11D-A219-01	0.729	0.229	-0.304	-0.684

TCGA-CU-A3QU-01A-11D-A22Y-01	0.797	0.336	-0.422	-0.842
TCGA-CU-A3YL-01A-11D-A22Y-01	0.473	0.069	-0.294	-0.851
TCGA-CU-A5W6-01A-11D-A288-01	0.841	0.365	-0.281	-0.887
TCGA-CU-A72E-01A-12D-A338-01	0.553	0.109	-0.270	-0.819
TCGA-DK-A1A3-01A-11D-A13V-01	0.482	0.062	-0.188	-0.519
TCGA-DK-A1A5-01A-11D-A13V-01	0.581	0.194	-0.242	-0.646
TCGA-DK-A1A6-01A-11D-A13V-01	0.557	0.056	-0.388	-0.881
TCGA-DK-A1A7-01A-11D-A13V-01	0.668	0.103	-0.172	-0.761
TCGA-DK-A1AA-01A-11D-A13V-01	0.984	0.435	-0.510	-1.018
TCGA-DK-A1AB-01A-11D-A13V-01	0.721	0.261	-0.224	-0.692
TCGA-DK-A1AC-01A-11D-A13V-01	0.600	0.010	-0.386	-0.822
TCGA-DK-A1AD-01A-11D-A13V-01	0.385	-0.180	-0.681	-1.262
TCGA-DK-A1AE-01A-11D-A13V-01	0.647	0.251	-0.242	-0.605
TCGA-DK-A1AF-01A-11D-A13V-01	0.618	0.214	-0.286	-0.609
TCGA-DK-A1AG-01A-11D-A13V-01	0.933	0.384	-0.521	-1.029
TCGA-DK-A2HX-01A-12D-A18E-01	0.847	0.176	-0.365	-0.696
TCGA-DK-A2I1-01A-11D-A17S-01	0.547	0.111	-0.212	-0.664
TCGA-DK-A2I2-01A-11D-A17S-01	0.530	0.029	-0.140	-0.455
TCGA-DK-A2I4-01A-11D-A219-01	0.444	0.121	-0.226	-0.614
TCGA-DK-A2I6-01A-12D-A18E-01	0.385	-0.092	-0.302	-0.584
TCGA-DK-A3IK-01A-32D-A219-01	0.561	0.182	-0.400	-0.868
TCGA-DK-A3IL-01A-11D-A20B-01	0.918	0.207	-0.277	-0.834
TCGA-DK-A3IM-01A-11D-A20B-01	0.419	-0.154	-0.647	-1.002
TCGA-DK-A3IN-01A-11D-A20B-01	0.932	0.334	-0.207	-0.602
TCGA-DK-A3IQ-01A-31D-A20B-01	0.440	0.109	-0.166	-0.505
TCGA-DK-A3IS-01A-21D-A219-01	1.106	0.427	-0.566	-0.856
TCGA-DK-A3IT-01A-31D-A20B-01	0.645	0.193	-0.267	-0.606
TCGA-DK-A3IU-01A-11D-A20B-01	0.496	0.068	-0.141	-0.707
TCGA-DK-A3IV-01A-22D-A219-01	0.710	0.194	-0.194	-0.703
TCGA-DK-A3WW-01A-22D-A23L-01	0.572	0.192	-0.179	-0.656
TCGA-DK-A3WX-01A-22D-A22Y-01	0.496	0.197	-0.109	-0.408
TCGA-DK-A3WY-01A-11D-A22Y-01	0.498	0.127	-0.293	-0.673
TCGA-DK-A3X1-01A-12D-A22Y-01	0.435	0.080	-0.251	-0.582
TCGA-DK-A3X2-01A-11D-A22Y-01	0.753	0.381	-0.369	-0.741
TCGA-DK-A6AV-01A-12D-A30D-01	0.625	0.148	-0.159	-0.570
TCGA-DK-A6AW-01A-11D-A30D-01	0.810	0.301	-0.410	-0.668
TCGA-DK-A6B0-01A-11D-A31K-01	0.772	0.174	-0.448	-0.641
TCGA-DK-A6B1-01A-12D-A30D-01	0.797	0.094	-0.374	-0.891
TCGA-DK-A6B2-01A-11D-A30D-01	0.538	0.061	-0.181	-0.536
TCGA-DK-A6B5-01A-11D-A31K-01	0.797	0.466	-0.244	-0.632
TCGA-DK-A6B6-01A-11D-A30D-01	0.573	0.202	-0.307	-0.759
TCGA-DK-AA6L-01A-11D-A390-01	0.579	0.110	-0.237	-0.826
TCGA-DK-AA6M-01A-11D-A390-01	0.657	0.205	-0.312	-0.659

TCGA-DK-AA6P-01A-11D-A390-01	0.777	0.324	-0.491	-1.008
TCGA-DK-AA6Q-01A-11D-A390-01	0.831	0.411	-0.203	-0.744
TCGA-DK-AA6R-01A-11D-A42D-01	0.445	-0.161	-0.548	-0.976
TCGA-DK-AA6S-01A-21D-A390-01	0.626	0.254	-0.319	-0.610
TCGA-DK-AA6T-01A-11D-A390-01	0.870	0.151	-0.358	-0.979
TCGA-DK-AA6U-01A-11D-A390-01	0.818	0.390	-0.231	-0.813
TCGA-DK-AA6W-01A-12D-A390-01	0.757	0.337	-0.163	-0.502
TCGA-DK-AA6X-01A-12D-A42D-01	0.450	0.143	-0.260	-0.511
TCGA-DK-AA71-01A-31D-A390-01	0.566	0.017	-0.338	-0.685
TCGA-DK-AA74-01A-11D-A390-01	0.539	0.087	-0.187	-0.308
TCGA-DK-AA75-01A-11D-A390-01	0.629	0.330	-0.445	-0.752
TCGA-DK-AA76-01A-11D-A390-01	0.993	0.404	-0.266	-0.832
TCGA-DK-AA77-01A-11D-A390-01	0.795	0.246	-0.303	-0.949
TCGA-E5-A2PC-01A-11D-A201-01	0.866	0.406	-0.434	-0.805
TCGA-E5-A4TZ-01A-11D-A31K-01	0.682	0.166	-0.375	-1.046
TCGA-E5-A4U1-01A-11D-A31K-01	0.634	0.101	-0.400	-0.892
TCGA-E7-A3X6-01A-12D-A22Y-01	0.698	0.278	-0.150	-0.740
TCGA-E7-A3Y1-01A-11D-A22Y-01	0.553	0.052	-0.150	-0.319
TCGA-E7-A4IJ-01A-31D-A26L-01	0.620	0.152	-0.397	-1.019
TCGA-E7-A4XJ-01A-11D-A26L-01	0.789	0.264	-0.503	-0.947
TCGA-E7-A519-01A-11D-A26L-01	0.706	0.464	-0.287	-0.828
TCGA-E7-A541-01A-11D-A26L-01	0.639	0.090	-0.128	-0.629
TCGA-E7-A5KE-01A-11D-A288-01	0.539	0.216	-0.212	-0.737
TCGA-E7-A5KF-01A-11D-A288-01	0.825	0.114	-0.532	-1.048
TCGA-E7-A677-01A-11D-A30D-01	0.790	0.184	-0.559	-1.067
TCGA-E7-A678-01A-11D-A30D-01	0.515	0.346	-0.365	-0.672
TCGA-E7-A6MD-01A-41D-A34T-01	0.660	0.127	-0.462	-0.890
TCGA-E7-A6ME-01A-22D-A32A-01	0.957	0.255	-0.399	-0.843
TCGA-E7-A6MF-01A-12D-A32A-01	0.804	0.182	-0.262	-0.803
TCGA-E7-A7DU-01A-11D-A32A-01	0.821	0.256	-0.431	-0.851
TCGA-E7-A7DV-01A-11D-A338-01	0.630	0.137	-0.275	-0.557
TCGA-E7-A7PW-01A-11D-A34T-01	0.735	0.114	-0.468	-0.799
TCGA-E7-A7XN-01A-11D-A34T-01	0.635	0.215	-0.350	-0.624
TCGA-E7-A85H-01A-11D-A34T-01	0.867	0.148	-0.223	-0.707
TCGA-E7-A8O7-01A-11D-A363-01	0.707	0.246	-0.335	-1.021
TCGA-E7-A97P-01A-11D-A38F-01	0.527	0.123	-0.224	-0.644
TCGA-E7-A97Q-01A-11D-A38F-01	0.486	0.300	-0.338	-0.951
TCGA-FD-A3B3-01A-12D-A201-01	0.306	0.047	-0.106	-0.211
TCGA-FD-A3B4-01A-12D-A201-01	0.578	0.166	-0.270	-0.795
TCGA-FD-A3B5-01A-11D-A20B-01	0.596	0.297	-0.099	-0.761
TCGA-FD-A3B6-01A-21D-A20B-01	0.540	0.039	-0.187	-0.801
TCGA-FD-A3B7-01A-31D-A20B-01	0.484	0.081	-0.162	-0.275
TCGA-FD-A3B8-01A-31D-A20B-01	0.442	0.175	-0.229	-0.430

TCGA-FD-A3N5-01A-11D-A219-01	0.567	0.139	-0.249	-0.733
TCGA-FD-A3N6-01A-11D-A219-01	0.940	0.197	-0.416	-1.022
TCGA-FD-A3NA-01A-11D-A219-01	0.411	-0.017	-0.267	-0.574
TCGA-FD-A3SJ-01A-12D-A22Y-01	0.614	0.056	-0.234	-0.993
TCGA-FD-A3SL-01A-21D-A22Y-01	0.492	0.128	-0.219	-0.614
TCGA-FD-A3SM-01A-11D-A22Y-01	0.847	0.201	-0.267	-0.824
TCGA-FD-A3SN-01A-12D-A22Y-01	0.628	0.410	-0.148	-0.608
TCGA-FD-A3SO-01A-11D-A22Y-01	0.775	0.210	-0.194	-0.590
TCGA-FD-A3SP-01A-31D-A22Y-01	0.416	0.045	-0.141	-0.254
TCGA-FD-A3SQ-01A-21D-A22Y-01	0.303	0.061	-0.076	-0.480
TCGA-FD-A3SR-01A-11D-A22Y-01	0.562	0.118	-0.512	-0.924
TCGA-FD-A3SS-01A-12D-A22Y-01	0.816	0.396	-0.282	-0.685
TCGA-FD-A43N-01A-11D-A23T-01	0.731	0.133	-0.279	-0.811
TCGA-FD-A43P-01A-31D-A23T-01	0.460	0.032	-0.242	-0.969
TCGA-FD-A43S-01A-21D-A23T-01	0.619	0.232	-0.326	-0.681
TCGA-FD-A43U-01A-11D-A23T-01	0.563	0.094	-0.148	-0.366
TCGA-FD-A43X-01A-11D-A23T-01	0.673	0.124	-0.345	-0.756
TCGA-FD-A43Y-01A-21D-A26L-01	0.657	0.140	-0.231	-0.578
TCGA-FD-A5BR-01A-11D-A26L-01	0.801	0.348	-0.306	-0.742
TCGA-FD-A5BS-01A-21D-A26L-01	0.651	0.240	-0.317	-0.640
TCGA-FD-A5BT-01A-11D-A26L-01	0.473	0.021	-0.254	-0.560
TCGA-FD-A5BU-01A-31D-A26L-01	0.565	0.153	-0.194	-0.541
TCGA-FD-A5BV-01A-11D-A26L-01	0.820	0.141	-0.383	-0.747
TCGA-FD-A5BX-01A-11D-A26L-01	0.960	0.144	-0.268	-0.849
TCGA-FD-A5BY-01A-31D-A288-01	0.859	0.406	-0.264	-0.724
TCGA-FD-A5BZ-01A-11D-A288-01	0.588	0.176	-0.365	-0.575
TCGA-FD-A5C0-01A-11D-A288-01	0.694	0.194	-0.509	-1.058
TCGA-FD-A5C1-01A-11D-A288-01	0.483	0.112	-0.227	-0.413
TCGA-FD-A62N-01A-11D-A30D-01	0.356	0.146	-0.064	-0.338
TCGA-FD-A62O-01A-11D-A30D-01	0.668	0.281	-0.365	-0.721
TCGA-FD-A62P-01A-32D-A30D-01	0.496	0.092	-0.206	-0.392
TCGA-FD-A62S-01A-11D-A30D-01	0.550	0.146	-0.120	-0.596
TCGA-FD-A6TA-01A-12D-A338-01	0.976	0.250	-0.122	-0.614
TCGA-FD-A6TB-01A-12D-A338-01	0.310	0.084	-0.094	-0.401
TCGA-FD-A6TC-01A-21D-A338-01	0.530	0.102	-0.245	-0.584
TCGA-FD-A6TD-01A-51D-A338-01	0.478	0.131	-0.136	-0.499
TCGA-FD-A6TE-01A-12D-A338-01	0.803	0.327	-0.319	-0.780
TCGA-FD-A6TF-01A-52D-A32A-01	0.505	0.174	-0.343	-0.569
TCGA-FD-A6TG-01A-11D-A32A-01	0.516	0.209	-0.332	-0.526
TCGA-FD-A6TH-01A-11D-A32A-01	0.471	0.067	-0.224	-0.886
TCGA-FD-A6TI-01A-11D-A32A-01	0.428	-0.040	-0.371	-0.815
TCGA-FD-A6TK-01A-42D-A338-01	0.754	0.181	-0.424	-0.788
TCGA-FJ-A3Z7-01A-12D-A23L-01	0.674	0.198	-0.254	-0.924

TCGA-FJ-A3Z9-01A-11D-A26L-01	0.698	0.286	-0.287	-0.634
TCGA-FJ-A3ZE-01A-11D-A23L-01	1.048	0.354	-0.550	-1.132
TCGA-FJ-A3ZF-01A-11D-A23L-01	0.719	0.226	-0.081	-0.614
TCGA-FJ-A871-01A-11D-A34T-01	0.490	0.191	-0.277	-0.528
TCGA-FT-A3EE-01A-11D-A201-01	0.557	-0.073	-0.589	-1.122
TCGA-FT-A61P-01A-11D-A30D-01	0.517	-0.048	-0.258	-0.556
TCGA-G2-A2EF-01A-12D-A18E-01	0.613	0.128	-0.485	-0.978
TCGA-G2-A2EJ-01A-11D-A17S-01	0.477	0.130	-0.355	-0.879
TCGA-G2-A2EK-01A-22D-A18E-01	0.659	0.093	-0.230	-0.577
TCGA-G2-A2EL-01A-12D-A18E-01	0.786	0.301	-0.280	-0.991
TCGA-G2-A2EO-01A-11D-A17S-01	0.474	0.022	-0.236	-0.511
TCGA-G2-A2ES-01A-11D-A17S-01	0.692	0.175	-0.245	-0.616
TCGA-G2-A3IB-01A-11D-A20B-01	0.783	0.331	-0.363	-0.977
TCGA-G2-A3IE-01A-11D-A20B-01	0.813	0.256	-0.309	-0.600
TCGA-G2-A3VY-01A-11D-A22Y-01	1.071	0.134	-0.899	-1.642
TCGA-G2-AA3B-01A-11D-A390-01	0.961	-0.234	-0.702	-1.122
TCGA-G2-AA3C-01A-21D-A390-01	0.659	0.255	-0.294	-0.778
TCGA-G2-AA3D-01A-11D-A390-01	0.691	0.062	-0.665	-0.996
TCGA-G2-AA3F-01A-12D-A42D-01	1.049	0.267	-0.427	-0.873
TCGA-GC-A3BM-01A-11D-A22Y-01	0.937	0.501	-0.258	-0.968
TCGA-GC-A3I6-01A-11D-A20B-01	0.635	0.021	-0.495	-0.996
TCGA-GC-A3OO-01A-11D-A22Y-01	0.487	0.108	-0.183	-0.417
TCGA-GC-A3RB-01A-12D-A21Y-01	0.821	0.135	-0.382	-0.705
TCGA-GC-A3RC-01A-11D-A22Y-01	0.541	0.161	-0.259	-0.541
TCGA-GC-A3RD-01A-12D-A22Y-01	0.759	0.194	-0.484	-1.001
TCGA-GC-A3WC-01A-31D-A22Y-01	0.375	0.133	-0.198	-0.441
TCGA-GC-A3YS-01A-11D-A23L-01	0.949	0.235	-0.334	-0.843
TCGA-GC-A4ZW-01A-11D-A26L-01	1.210	0.209	-0.599	-1.632
TCGA-GC-A6I1-01A-12D-A31K-01	0.388	0.081	-0.089	-0.315
TCGA-GC-A6I3-01A-11D-A31K-01	0.539	0.038	-0.172	-0.713
TCGA-GD-A2C5-01A-12D-A17S-01	0.751	0.114	-0.266	-0.815
TCGA-GD-A3OP-01A-21D-A21Y-01	0.413	0.066	-0.152	-0.338
TCGA-GD-A3OQ-01A-32D-A21Y-01	0.331	0.088	-0.146	-0.517
TCGA-GD-A3OS-01A-12D-A21Y-01	0.622	0.041	-0.670	-1.057
TCGA-GD-A6C6-01A-21D-A31K-01	1.327	0.390	-0.773	-1.467
TCGA-GD-A76B-01A-11D-A32A-01	0.416	0.117	-0.150	-0.400
TCGA-GU-A42P-01A-11D-A23T-01	0.459	0.031	-0.179	-0.809
TCGA-GU-A42Q-01A-11D-A23T-01	0.502	0.146	-0.104	-0.427
TCGA-GU-A42R-01A-11D-A23L-01	0.528	0.229	-0.247	-0.748
TCGA-GU-A762-01A-11D-A338-01	0.486	0.147	-0.200	-0.499
TCGA-GU-A763-01A-11D-A32A-01	0.666	0.310	-0.513	-1.240
TCGA-GU-A764-01A-11D-A34T-01	0.613	0.153	-0.219	-0.420
TCGA-GU-A766-01A-11D-A32A-01	0.386	0.120	-0.155	-0.348

TCGA-GU-A767-01A-11D-A32A-01	0.651	0.264	-0.189	-0.479
TCGA-GU-AATO-01A-11D-A390-01	0.679	0.114	-0.580	-1.032
TCGA-GU-AATP-01A-11D-A390-01	0.696	0.345	-0.228	-0.661
TCGA-GU-AATQ-01A-11D-A390-01	0.851	0.064	-0.385	-0.833
TCGA-GV-A3JV-01A-11D-A21Y-01	0.637	0.047	-0.316	-0.582
TCGA-GV-A3JW-01A-11D-A20B-01	0.520	0.246	-0.689	-1.176
TCGA-GV-A3JX-01A-11D-A20B-01	0.782	0.281	-0.526	-0.874
TCGA-GV-A3JZ-01A-11D-A219-01	0.706	0.117	-0.368	-0.723
TCGA-GV-A3QF-01A-31D-A22Y-01	0.449	0.069	-0.237	-0.576
TCGA-GV-A3QG-01A-11D-A21Y-01	0.661	0.055	-0.324	-0.615
TCGA-GV-A3QH-01A-11D-A21Y-01	0.614	0.203	-0.419	-1.033
TCGA-GV-A3QI-01A-11D-A21Y-01	0.700	0.175	-0.325	-0.794
TCGA-GV-A40E-01A-12D-A23L-01	0.578	0.273	-0.276	-0.769
TCGA-GV-A40G-01A-11D-A23L-01	0.894	0.240	-0.813	-1.358
TCGA-GV-A6ZA-01A-12D-A338-01	0.598	0.187	-0.298	-0.766
TCGA-H4-A2HO-01A-11D-A17S-01	0.556	0.160	-0.333	-0.639
TCGA-H4-A2HQ-01A-11D-A17S-01	0.994	-0.056	-0.654	-1.267
TCGA-HQ-A2OE-01A-11D-A201-01	1.190	0.367	-0.925	-1.781
TCGA-HQ-A2OF-01A-11D-A26L-01	0.665	-0.062	-0.853	-1.419
TCGA-HQ-A5ND-01A-11D-A26L-01	0.551	0.066	-0.265	-0.709
TCGA-HQ-A5NE-01A-12D-A288-01	0.514	0.118	-0.350	-0.552
TCGA-K4-A3WS-01A-11D-A22Y-01	0.403	0.177	-0.146	-0.461
TCGA-K4-A3WV-01A-11D-A22Y-01	0.834	0.172	-0.748	-1.362
TCGA-K4-A4AC-01A-21D-A26L-01	0.477	0.138	-0.282	-0.556
TCGA-K4-A54R-01A-11D-A26L-01	0.700	0.328	-0.350	-0.721
TCGA-K4-A5RH-01A-11D-A30D-01	0.425	0.070	-0.391	-0.641
TCGA-K4-A5RI-01A-11D-A288-01	0.593	0.100	-0.417	-0.707
TCGA-K4-A5RJ-01A-11D-A288-01	0.729	0.277	-0.256	-0.789
TCGA-K4-A6FZ-01A-11D-A31K-01	0.577	0.157	-0.416	-0.675
TCGA-K4-A6MB-01A-11D-A31K-01	0.521	0.093	-0.271	-0.658
TCGA-K4-A83P-01A-11D-A34T-01	0.241	0.079	-0.195	-0.381
TCGA-K4-AAQO-01A-11D-A38F-01	0.578	0.109	-0.351	-0.601
TCGA-KQ-A41N-01A-11D-A338-01	0.616	0.228	-0.450	-0.741
TCGA-KQ-A41O-01A-12D-A34T-01	0.860	0.263	-0.529	-1.352
TCGA-KQ-A41P-01A-12D-A338-01	0.445	0.171	-0.346	-0.927
TCGA-KQ-A41Q-01A-11D-A338-01	0.633	0.181	-0.134	-0.489
TCGA-KQ-A41R-01A-21D-A34T-01	0.672	0.219	-0.515	-0.927
TCGA-KQ-A41S-01A-12D-A338-01	0.505	0.086	-0.488	-0.738
TCGA-LC-A66R-01A-41D-A30D-01	0.569	0.020	-0.246	-0.512
TCGA-LT-A5Z6-01A-11D-A288-01	0.754	0.358	-0.328	-0.901
TCGA-LT-A8JT-01A-11D-A363-01	0.692	0.248	-0.422	-0.923
TCGA-MV-A51V-01A-11D-A26L-01	1.305	0.659	-0.487	-0.859
TCGA-PQ-A6FI-01A-11D-A31K-01	0.764	0.328	-0.188	-0.552

TCGA-PQ-A6FN-01A-11D-A31K-01	0.324	0.130	-0.169	-0.371
TCGA-R3-A69X-01A-22D-A30D-01	0.452	0.145	-0.307	-0.598
TCGA-S5-A6DX-01A-11D-A31K-01	0.435	0.063	-0.211	-0.744
TCGA-S5-AA26-01A-11D-A38F-01	0.760	0.245	-0.581	-1.146
TCGA-SY-A9G0-01A-12D-A38F-01	0.314	0.112	-0.154	-0.307
TCGA-SY-A9G5-01A-11D-A38F-01	0.429	0.147	-0.281	-0.451
TCGA-UY-A78K-01A-11D-A338-01	0.520	0.068	-0.166	-0.610
TCGA-UY-A78L-01A-12D-A338-01	0.387	0.048	-0.243	-0.735
TCGA-UY-A78M-01A-21D-A34T-01	0.480	0.028	-0.335	-0.731
TCGA-UY-A78N-01A-12D-A338-01	0.632	0.123	-0.296	-0.676
TCGA-UY-A78O-01A-12D-A338-01	0.572	0.200	-0.091	-0.728
TCGA-UY-A78P-01A-12D-A363-01	0.253	0.099	-0.159	-0.345
TCGA-UY-A8OB-01A-12D-A42D-01	0.876	0.295	-0.456	-0.989
TCGA-UY-A8OC-01A-11D-A363-01	0.544	0.036	-0.170	-0.740
TCGA-UY-A8OD-01A-11D-A363-01	1.063	0.409	-0.378	-0.814
TCGA-UY-A9PA-01A-11D-A38F-01	0.424	0.133	-0.190	-0.480
TCGA-UY-A9PB-01A-11D-A38F-01	0.672	0.131	-0.168	-0.604
TCGA-UY-A9PD-01A-11D-A38F-01	0.445	-0.007	-0.387	-0.726
TCGA-UY-A9PE-01A-11D-A38F-01	0.685	0.257	-0.251	-0.574
TCGA-UY-A9PF-01A-11D-A38F-01	0.480	0.149	-0.263	-0.844
TCGA-UY-A9PH-01A-11D-A38F-01	0.676	0.289	-0.236	-0.575
TCGA-XF-A8HB-01A-11D-A363-01	1.060	0.230	-0.497	-1.298
TCGA-XF-A8HC-01A-11D-A363-01	1.338	0.272	-0.858	-1.617
TCGA-XF-A8HD-01A-11D-A363-01	0.473	0.223	-0.245	-0.463
TCGA-XF-A8HE-01A-11D-A363-01	0.599	0.211	-0.152	-0.418
TCGA-XF-A8HF-01A-11D-A363-01	0.643	0.215	-0.439	-0.730
TCGA-XF-A8HG-01A-11D-A363-01	0.825	0.227	-0.596	-1.113
TCGA-XF-A8HH-01A-11D-A38F-01	0.534	0.146	-0.314	-0.637
TCGA-XF-A8HI-01A-11D-A38F-01	0.737	0.293	-0.385	-1.031
TCGA-XF-A9SG-01A-12D-A42D-01	0.719	0.291	-0.169	-0.686
TCGA-XF-A9SH-01A-11D-A390-01	0.658	0.157	-0.327	-0.925
TCGA-XF-A9SI-01A-11D-A390-01	0.397	0.066	-0.152	-0.281
TCGA-XF-A9SJ-01A-11D-A390-01	0.619	0.280	-0.318	-0.778
TCGA-XF-A9SK-01A-11D-A42D-01	0.611	0.078	-0.148	-0.503
TCGA-XF-A9SL-01A-11D-A390-01	0.255	0.093	-0.165	-0.423
TCGA-XF-A9SM-01A-11D-A42D-01	0.543	0.115	-0.183	-0.401
TCGA-XF-A9SP-01A-11D-A390-01	0.767	0.186	-0.234	-0.589
TCGA-XF-A9ST-01A-11D-A42D-01	0.564	0.209	-0.679	-0.946
TCGA-XF-A9SU-01A-31D-A390-01	0.464	0.133	-0.158	-0.448
TCGA-XF-A9SV-01A-21D-A42D-01	0.517	0.210	-0.113	-0.517
TCGA-XF-A9SW-01A-11D-A42D-01	0.422	0.155	-0.103	-0.297
TCGA-XF-A9SX-01A-21D-A390-01	0.462	0.236	-0.248	-0.668
TCGA-XF-A9SY-01A-21D-A42D-01	0.476	0.153	-0.227	-0.453

TCGA-XF-A9SZ-01A-11D-A390-01	0.751	0.242	-0.315	-0.549
TCGA-XF-A9T0-01A-11D-A390-01	0.834	0.205	-0.474	-0.877
TCGA-XF-A9T2-01A-11D-A42D-01	0.480	0.189	-0.247	-0.586
TCGA-XF-A9T3-01A-11D-A42D-01	0.703	0.121	-0.428	-0.686
TCGA-XF-A9T4-01A-11D-A390-01	0.607	0.228	-0.330	-0.669
TCGA-XF-A9T5-01A-11D-A42D-01	0.676	0.102	-0.527	-0.842
TCGA-XF-A9T6-01A-11D-A42D-01	0.695	0.154	-0.339	-0.912
TCGA-XF-A9T8-01A-11D-A390-01	0.566	0.081	-0.145	-0.573
TCGA-XF-AAME-01A-12D-A42D-01	0.326	0.068	-0.118	-0.304
TCGA-XF-AAMF-01A-21D-A42D-01	0.812	0.263	-0.189	-0.722
TCGA-XF-AAMG-01A-11D-A42D-01	1.034	0.226	-0.581	-1.534
TCGA-XF-AAMH-01A-11D-A42D-01	0.914	0.123	-0.378	-0.959
TCGA-XF-AAMJ-01A-11D-A42D-01	0.491	0.193	-0.147	-0.332
TCGA-XF-AAML-01A-11D-A42D-01	0.705	0.295	-0.415	-0.748
TCGA-XF-AAMQ-01A-11D-A42D-01	0.692	0.062	-0.342	-0.833
TCGA-XF-AAMR-01A-31D-A42D-01	0.413	0.114	-0.209	-0.515
TCGA-XF-AAMT-01A-11D-A42D-01	0.678	0.420	-0.436	-0.662
TCGA-XF-AAMW-01A-11D-A42D-01	0.555	0.313	-0.354	-0.668
TCGA-XF-AAMX-01A-11D-A42D-01	0.958	0.049	-0.568	-1.222
TCGA-XF-AAMY-01A-11D-A42D-01	0.753	0.208	-0.349	-0.664
TCGA-XF-AAMZ-01A-11D-A42D-01	0.763	0.302	-0.400	-0.836
TCGA-XF-AAN0-01A-11D-A42D-01	0.795	0.359	-0.331	-0.731
TCGA-XF-AAN1-01A-31D-A42D-01	0.715	0.266	-0.351	-1.260
TCGA-XF-AAN2-01A-11D-A42D-01	0.605	0.209	-0.259	-0.647
TCGA-XF-AAN3-01A-11D-A42D-01	0.670	0.129	-0.323	-0.638
TCGA-XF-AAN4-01A-11D-A42D-01	0.467	0.104	-0.139	-0.365
TCGA-XF-AAN5-01A-11D-A42D-01	0.450	0.143	-0.349	-0.608
TCGA-XF-AAN7-01A-11D-A42D-01	0.872	0.339	-0.363	-0.646
TCGA-XF-AAN8-01A-11D-A42D-01	0.457	0.158	-0.181	-0.472
TCGA-YC-A89H-01A-11D-A363-01	0.859	0.068	-0.368	-0.780
TCGA-YC-A8S6-01A-31D-A38F-01	0.401	0.086	-0.221	-0.455
TCGA-YC-A9TC-01A-22D-A390-01	0.544	0.107	-0.288	-0.789
TCGA-YF-AA3L-01A-11D-A38F-01	0.953	0.316	-0.468	-0.823
TCGA-YF-AA3M-01A-11D-A42D-01	0.823	0.185	-0.348	-0.727
TCGA-ZF-A9R0-01A-11D-A38F-01	0.654	0.250	-0.339	-0.735
TCGA-ZF-A9R1-01A-11D-A390-01	0.850	0.285	-0.207	-0.619
TCGA-ZF-A9R2-01A-11D-A390-01	0.671	0.275	-0.419	-0.572
TCGA-ZF-A9R3-01A-11D-A38F-01	0.528	0.108	-0.377	-0.869
TCGA-ZF-A9R4-01A-11D-A38F-01	1.113	0.435	-0.470	-0.970
TCGA-ZF-A9R5-01A-12D-A42D-01	0.609	0.238	-0.376	-0.909
TCGA-ZF-A9R7-01A-11D-A38F-01	0.542	0.137	-0.330	-0.701
TCGA-ZF-A9R9-01A-11D-A38F-01	0.971	0.277	-0.636	-0.918
TCGA-ZF-A9RC-01A-11D-A38F-01	0.829	0.191	-0.326	-0.471

TCGA-ZF-A9RD-01A-11D-A42D-01	0.726	0.104	-0.154	-0.428
TCGA-ZF-A9RE-01A-11D-A38F-01	0.826	0.326	-0.344	-0.821
TCGA-ZF-A9RF-01A-11D-A38F-01	0.791	0.145	-0.582	-0.873
TCGA-ZF-A9RG-01A-21D-A42D-01	0.439	0.124	-0.248	-0.385
TCGA-ZF-A9RL-01A-11D-A38F-01	0.608	0.220	-0.571	-0.862
TCGA-ZF-A9RM-01A-11D-A38F-01	0.777	0.350	-0.434	-0.958
TCGA-ZF-A9RN-01A-11D-A42D-01	1.086	0.311	-0.545	-1.078
TCGA-ZF-AA4N-01A-11D-A38F-01	0.805	0.143	-0.277	-0.826
TCGA-ZF-AA4R-01A-11D-A38F-01	0.715	0.158	-0.545	-0.754
TCGA-ZF-AA4T-01A-11D-A38F-01	0.672	0.195	-0.628	-0.822
TCGA-ZF-AA4U-01A-11D-A38F-01	0.698	0.229	-0.546	-0.933
TCGA-ZF-AA4V-01A-11D-A38F-01	0.736	-0.056	-0.435	-0.839
TCGA-ZF-AA4W-01A-12D-A38F-01	0.386	0.127	-0.196	-0.325
TCGA-ZF-AA4X-01A-11D-A38F-01	0.684	0.450	-0.366	-0.899
TCGA-ZF-AA51-01A-21D-A390-01	0.769	0.447	-0.481	-0.830
TCGA-ZF-AA52-01A-12D-A390-01	0.529	0.068	-0.150	-0.578
TCGA-ZF-AA53-01A-11D-A390-01	0.568	0.132	-0.166	-0.562
TCGA-ZF-AA54-01A-11D-A390-01	0.800	0.105	-0.476	-0.742
TCGA-ZF-AA56-01A-31D-A390-01	0.476	0.056	-0.170	-0.662
TCGA-ZF-AA58-01A-12D-A42D-01	0.569	0.141	-0.246	-0.424
TCGA-ZF-AA5H-01A-11D-A390-01	0.656	0.260	-0.523	-0.927
TCGA-ZF-AA5N-01A-11D-A42D-01	1.024	0.232	-0.381	-0.882
TCGA-ZF-AA5P-01A-11D-A390-01	0.406	0.091	-0.191	-0.377

Supplementary Table S4. Frequently altered genomic regions in the TCGA bladder cancer cohort (n=400). Genomic regions gained in >100 samples were considered as frequent gains. Genomic regions lost in >80 samples were considered as frequent losses. The frequent genomic alterations are listed for **A**) gains and **B**) losses.

A) Gains

Chromosome	Start	End	Genomic band	Length	Indicator[1]	Name in graphs
chr1	150563315	151552479	1q21	989164	region	1q21
chr1	160996755	161169165	1q23	172410	region	1q23
chr2	61204857	64298416	2p16-p14	3093559	region	2p16-p14
chr2	16080683	16087129	2p24	6446	*MYCN*	2p24_(*MYCN*)
chr2	9724106	9771106	2p25	47000	*YWHAQ*	2p25_(*YWHAQ*)
chr3	12393001	12475855	3p25	82854	*PPARG*	3p25_(*PPARG*/*RAF1*)
chr3	178254224	178562217	3q26-q29	307993	*KCNMB2*	3q26-q29_(*KCNMB2*)
chr5	1253287	1295162	5p	41875	*TERT*	5p_(*TERT*)
chr6	20368247	21899443	6p22	1531196	region	6p22_(*E2F3*/*CDKAL1*/*SOX4*)
chr7	1000000	20012474	7p22-p21	19012474	large region	7p22-p21
chr8	101930804	101965221	8q22	34417	*YWHAZ*	8q22_(*YWHAZ*)
chr8	128748315	128753680	8q24	5365	*MYC*	8q24_(*MYC*)
chr10	8096667	8117164	10p15-p14	20497	*GATA3*	10p15-p14_(*GATA3*)
chr11	69455873	69469242	11q13	13369	*CCND1*	11q13_(*CCND1*)
chr12	2966847	2986321	12p13	19474	*FOXM1*	12p13_(*FOXM1*)
chr12	69201971	69239320	12q15	37349	*MDM2*	12q15_(*MDM2*)
chr13	73633142	73651676	13q21	18534	*KLF5*	13q21_(*KLF5*)
chr17	26759573	27604619	17q11	845046	region	17q11
chr17	37856254	37884915	17q12	28661	*ERBB2*	17q12_(*ERBB2*)
chr17	60603643	80917016	17q23-q25	20313373	large region	17q23-q25
chr18	329586	4819344	18p11	4489758	region	18p11
chr19	32500000	58878226	19q	26378226	large region	19q
chr20	29843974	34285482	20q11	4441508	region	20q11

B) Losses

Chromosome	Start	End	Genomic band	Length	Indicator[1]	Name in graphs
chr2	225098795	226326879	2q36	1228084	region	2q36
chr2	233080468	234584324	2q37	1503856	region	2q37
chr5	52000000	176000000	5q	124000000	large region	5q
chr6	110654419	112320530	6q21	1666111	region	6q21
chr6	157099064	157531913	6q25	432849	*ARID1B*	6q25_(*ARID1B*)
chr8	1000000	38000000	8p	37000000	large region	8p
chr9	21967751	21975132	9p21	7381	*CDKN2A*	9p21_(*CDKN2A*)
chr9	65000000	140000000	9q	75000000	large region	9q
chr10	89623195	89728532	10q23	105337	*PTEN*	10q23_(*PTEN*)
chr10	102505468	102589698	10q24-q25	84230	*PAX2*	10q24-q25_(*PAX2*)
chr11	1000000	48000000	11p	47000000	large region	11p
chr13	48877883	49056026	13q14	178143	*RB1*	13q14_(*RB1*)
chr16	2705764	4563157	16p13	1857393	region	16p13
chr17	7571720	7590868	17p13	19148	*TP53*	17p13_(*TP53*)
chr18	48556583	48611411	18q21	54828	*SMAD4*	18q21_(*SMAD4*)
chr22	40900078	41588938	22q13	688860	region	22q13

1) Indicator. Based on the observed alterations in the cohort, frequently altered regions were labeled as Gene (<1Mbp), Small region (<5Mbp), or Large region (>5Mbp). Gene regions were <1Mbp and is indicated by the affected gene in the table. In each sample, alterations should cover the full gene when amplified, or cover at least 50% when deleted. For “Small regions” (<5Mbp), 75% of the region should be altered to call a gain or loss. For “Large regions” (>5Mbp) 50% of the region should be altered to call a gain or loss.

References:

1. Sjödahl, G., Eriksson, P., Liedberg, F. & Höglund, M. Molecular classification of urothelial carcinoma: global mRNA classification versus tumour-cell phenotype classification. *J. Pathol.* **242,** 113–125 (2017).
2. Dabney, A. R. Classification of microarrays to nearest centroids. *Bioinforma. Oxf. Engl.* **21,** 4148–4154 (2005).
3. Heyer, L. J., Kruglyak, S. & Yooseph, S. Exploring Expression Data: Identification and Analysis of Coexpressed Genes. *Genome Res.* **9,** 1106–1115 (1999).
